# Emergence and Spread of Cephalosporinases in Wildlife: A Review

**DOI:** 10.3390/ani11061765

**Published:** 2021-06-12

**Authors:** Josman D. Palmeira, Mónica V. Cunha, João Carvalho, Helena Ferreira, Carlos Fonseca, Rita T. Torres

**Affiliations:** 1 Departamento de Biologia & CESAM, Universidade de Aveiro, Campus de Santiago, 3810-193 Aveiro, Portugal; josmandantasp@gmail.com (J.D.P.); jlocarvalho@gmail.com (J.C.); cfonseca@ua.pt (C.F.); 2 Centre for Ecology, Evolution and Environmental Changes (cE3c), Faculdade de Ciências, Universidade de Lisboa, 1749-016 Lisbon, Portugal; mscunha@fc.ul.pt; 3 Biosystems & Integrative Sciences Institute (BioISI), Faculdade de Ciências, Universidade de Lisboa, 1749-016 Lisbon, Portugal; 4 Microbiology, Department of Biological Sciences, Faculty of Pharmacy, University of Porto, 4050-313 Porto, Portugal; hferr@ff.up.pt; 5 UCIBIO, REQUIMTE, University of Porto, 4050-313 Porto, Portugal; 6 ForestWISE-Collaborative Laboratory for Integrated Forest & Fire Management, Quinta de Prados, 5001-801 Vila Real, Portugal

**Keywords:** one health, wildlife, cephalosporinases, ESBL, AmpC, CTX-M

## Abstract

**Simple Summary:**

Antimicrobial resistance (AMR) is one of the global public health challenges nowadays. AMR threatens the effective prevention and treatment of an ever-increasing range of infections, being present in healthcare settings but also detected across the whole ecosystem, including wildlife. This work compiles the available information about an important resistance mechanism that gives bacteria the ability to inactivate cephalosporin antibiotics, the cephalosporinases (extended-spectrum beta-lactamase (ESBL) and AmpC beta-lactamase), in wildlife. Through a rigorous systematic literature review in the Web of Science database, the available publications on this topic in the wildlife sphere were analysed. The emergence and spread of cephalosporinases in wildlife has been reported in 46 countries from all continents (52% in Europe), with descriptions mainly in birds and mammals. The most widely disseminated cephalosporinases in human-related settings (e.g. CTX-M-1, CTX-M-14, CTX-M-15 and CMY-2) are also the most reported in wildlife, suggesting that anthropogenic pressure upon natural environments have a strong impact on antimicrobial resistance spread, including the dissemination of genes encoding these enzymes. Our work highlights the urgence and importance of public and ecosystem health policies, including improved surveillance and control strategies that breakdown AMR transmission chains across wildlife, as part of an integrated strategy of the One Health approach.

**Abstract:**

In the last decade, detection of antibiotic resistant bacteria from wildlife has received increasing interest, due to the potential risk posed by those bacteria to wild animals, livestock or humans at the interface with wildlife, and due to the ensuing contamination of the environment. According to World Health Organization, cephalosporins are critically important antibiotics to human health. However, acquired resistance to β-lactams is widely distributed and is mainly mediated by extended-spectrum beta-lactamase and AmpC beta-lactamases, such as cephalosporinases. This work thus aimed to compile and analyse the information available on the emergence and dissemination of cephalosporinases in wildlife worldwide. Results suggest a serious scenario, with reporting of cephalosporinases in 46 countries from all continents (52% in Europe), across 188 host species, mainly birds and mammals, especially gulls and ungulates. The most widely reported cephalosporinases, CTX-M-1, CTX-M-14, CTX-M-15 and CMY-2, were also the most common in wild animals, in agreement with their ubiquity in human settings, including their association to high-risk clones of *Escherichia coli* (*E. coli*), such as the worldwide distributed CTX-M-15/ST131 *E. coli*. Altogether, our findings show that anthropogenic activities affect the whole ecosystem and that public policies promoting animal and environmental surveillance, as well as mitigation measures to avoid antimicrobial misuse and AMR spread, are urgently needed to be out in practise.

## 1. Introduction

*Enterobacteriaceae* are Gram-negative bacteria naturally inhabiting the intestinal tract of mammals and are among the most common human opportunistic pathogens, being responsible for a myriad of nosocomial and community-acquired infections (e.g., gastroenteritis, urinary tract infections, meningitis, pneumonia, septicaemia, among others) [1]. Over the years, many *Enterobacteriaceae* have become resistant to commonly used antimicrobial compounds and antimicrobial resistance among these organisms is an emerging problem. Among the several therapeutic options to treat the infections caused by these microorganisms, β-lactams (penicillin, cephalosporins and carbapenems) are the most common group of antibiotics prescribed due to their effective and broad spectrum of action on different bacterial pathogens, combined with their low toxicity in humans and animals [2]. Acquired resistance to β-lactams is widely distributed and mainly mediated by β-lactamases, such as cephalosporinase (extended-spectrum β-lactamases (ESBL) and AmpC beta-lactamases). Cephalosporinases confer bacterial resistance to most β-lactams, including oxyimino-β-lactam antibiotics [2]. Antibiotic resistance among opportunistic and/or commensal pathogens is rapidly rising globally, hampering the treatment of infections and increasing morbidity, mortality and health care costs [3,4]. Since 2000, the spread of community-acquired ESBL-producing bacteria, mainly *Escherichia coli*, has been reported worldwide [5]. The cephalosporins, a class of β-lactam antibiotics, are crucial for preventing and treating life-threatening nosocomial infections, which are often associated with medical procedures (transplantation, catheterization, intensive care admission, highly technical surgery). Of particular concern is the increased incidence of infections caused by *E. coli* isolates producing ESBLs, which has rendered the use of third generation cephalosporins increasingly ineffective against this pathogen [5]. AmpC beta-lactamases were initially classified as chromosomal beta-lactamases, but since the 1990s they have been recognized as important plasmid-mediated cephalosporinases related with therapeutical failures in community-acquired infections, especially associated with *E. coli*, *Klebsiella pneumonia* and *Salmonella enterica* infections [6].

The World Health Organization has deemed third, fourth, and fifth-generation cephalosporins to be critically important antibiotics [7], with the highest priority meaning that they i) should only be used to treat serious human infections and ii) are used to treat bacterial infections whose etiological agents may spill over from nonhuman sources [6]. Despite its prioritization, cephalosporin is the second largest antibiotic class used in infection treatment of humans [8] and food-producing animals [9]. For example, a third-generation cephalosporin, ceftiofur, is the most widely used antibiotic in U.S.A. for the treatment of common infections in cattle [9]. As the food chain is considered the main route of transmission of antimicrobial resistant determinants between commensals of animal and human populations [10], it is expected that the routine use of ceftiofur in livestock production could increase the risk of zoonotic transmission of β-lactamase-encoding genes, as well as of other genes conferring resistance to critically important antimicrobials in public health [9]. The emergence of multidrug resistance in *Enterobacteriaceae* from animal sources is thus of utmost importance, due to the cascade effects with potential impacts on human clinical therapy. The opposite way is relevant too, but perhaps not by close contact with livestock, but by human usage of cephalosporins, which can proportionally be up to four times high than livestock and can have a disseminating impact on environmental via residual contamination. The influence of wildlife on the persistence and spread of antibiotic resistance determinants is often overlooked, namely those relevant mechanisms relating with beta-lactamase production [11]. Wildlife do not normally come into contact with antibiotics, but their association, both direct and indirect, with humans, livestock, domestic animals or humanized-environments, can enhance their contact with selective agents, commensals from humans and other species, as well as with bacterial resistance genes [12]. However, in the last decade, antibiotic resistant bacteria from natural reservoirs and wildlife have received increasing interest from the scientific community [12] and the potential risk they exert on the ensuing contamination of the environment has been acknowledged [12].

Extended-spectrum-cephalosporinase-producing *Enterobacteriaceae* have been widely reported worldwide from humans, livestock, companion animals and environmental sources [13]. Dissemination of resistance to cephalosporins considering the most relevant mechanisms, cephalosporinases (ESBL and AmpC beta-lactamases), is one of the emerging problems that we presently face in the global spread of AMR. Thus, studies setting a global picture of this problem are urgently needed to understand the dynamics of dissemination [14]. To fill this caveat, this work aims to compile and analyze the information available on the emergence and evolution of cephalosporinases in wildlife worldwide, prompting us to understand the role of this natural compartment on the spread and dissemination of ESBL and AmpC beta-lactamases.

## 2. Materials and Methods

### Search Strategy 

A systematic literature review of the available publications describing the presence of cephalosporinases in wildlife was performed using a rigorous search strategy in the online version of the Science Citation Index Expanded (SCI-EXPANDED) from the Web of Science (WoS) database (http://www.isiknowledge.com) [15]. We made no geographic, temporal or language restrictions on the search and the searches were updated on 6 July 2020. All available titles and abstracts were reviewed. The focus of this search was to find publications that contained both the main subjects of the study, cephalosporinases and wild animals. For this, an advanced search was performed, using the option of all databases and the following field tags, Boolean operators and keywords: For cephalosporinases, TS=((((((((ESBL OR “Extended-Spectrum β-Lactamase”) OR “Extended-Spectrum Beta-Lactamase”) OR CTX-M) OR SHV) OR TEM) OR Cephalosporinase) OR AmpC) OR CMY); for wild animals, TS=((((((((Boar OR Deer) OR “Wild Mammals”) OR Feral) OR “Wild Animals”) OR Wildlife) OR “Wild Birds”) OR Seagulls) OR Gulls). These two searches were combined by the Boolean operator “AND” to obtain only the intersection.

All the publications from literature search were analysed in two steps: (i) firstly, titles and abstracts were analysed and the publications which did not fill the inclusion criteria (e.g., focus on livestock, non-AMR description, etc.) were excluded; and (ii) secondly, the full text was analysed and the relevant information was extracted. All query results were verified manually before excluding duplicates (Figure S1).

Finally, results for all articles were imported into a bibliographic referencing tool (Mendeley Desktop 1.19.8). All publications were included with the following variables extracted: year of publication, location of analysed samples, animal data, bacterial data, cephalosporinase data, and citation.

A MLST-based minimal spanning tree was calculated and generated, using the goeBURST full MLST algorithm in Phyloviz 2.0, with the sequence typing number of ESBL- and AmpC-producing *E. coli* described in all evaluated publications.

## 3. Results and Discussion

From the literature search, a total of 1980 publications were obtained (Figure S1). This number was reduced to 215 after the first step of analysis (title and abstract) and, after the second step of analysis (full text), only 149 publications remained.

### 3.1. Temporal and Geographical Research Trends 

Publications focusing on cephalosporinases on wildlife are relatively recent and date from 2004. The first publication, in 2004, reported CMY-2-producing *Salmonella* isolated from wild boar (*Sus scrofa*) which had been imported from Canada to Denmark [14]. This single example highlights the importance of the international trade of live animals as a pathway for spreading resistant and pathogenic organisms. Unfortunately, mitigation measures to the effects of international trade on dispersion of AMR have been left out several National AMR Action Plans, jeopardizing global AMR control strategies [16].

**Figure 1:**
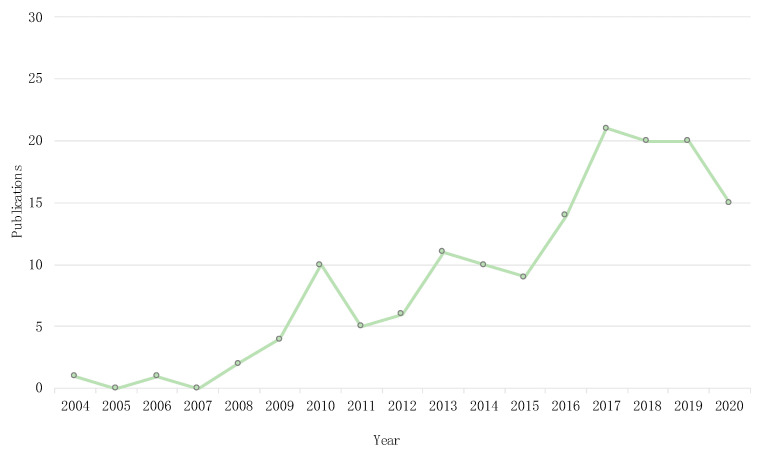
Trends of publication of cephalosporinases reported in wildlife since its first description in 2004.

The number of publications on this topic has grown since 2004 until now, but the largest number of publications, over 60%, is concentrated in the last 5 years (2016–2020) (Figure 1).

This increase was expected as it follows the increase trend of publications of AMR in wildlife [12]. The relevance of publications focusing on cephalosporinase detection in wildlife started with the discussion whether bacterial resistance in wildlife could be related with human antibiotic use, particularly because cephalosporins are important therapeutic options for serious infections at the healthcare setting level and are unusually used at the community level [17,18]. Since then, research has been focusing on untangling the routes of transmission of AMR, including cephalosporin resistance, between humans and wildlife, reinforcing the idea that the same antimicrobial resistance patterns co-occur in wildlife, livestock, and human populations.

Regarding the distribution of publications per continent, our results show a geographical bias, meaning that some continents stand out when compared with others. There were more publications describing the prevalence of cephalosporinase-producing bacteria (CB) in wildlife in Europe, with 54.4% of publications, followed by South America (14.1%), and North America (10.7%) (Table 1). We were expecting that Europe would stand out, as the bulk of AMR publications in wildlife are from this continent [12]. However, it is surprising that south America surpass the United States, mainly because USA is one of the most productive countries in AMR research in wildlife. However, when we focus on a specific mechanism of AMR, the publications rates are influenced not only by the proportion of scientific production, but also by the features of countries and/or work types. In this case, the more productive South American country, Brazil, shows characteristics that may explain the second place in the publications ranking. Brazil is one of the largest food-animal producers worldwide, with a continued need to improve production practices at the level of antibiotic usage, control, and sanitation.

During the late 1990s and early 2000s, the European Union (EU) implemented more rigorous regulations on the use of antimicrobial substances in animal production, particularly for growth promotion. The EU prevention strategy and combat of AMR finally led in 1998 to the launching of the European Antimicrobial Resistance Surveillance System (EARS-Net). While the previous initiatives were focused on food-production animals and humans, this alarmed the scientific community of the importance of One Health.

**Figure 2:**
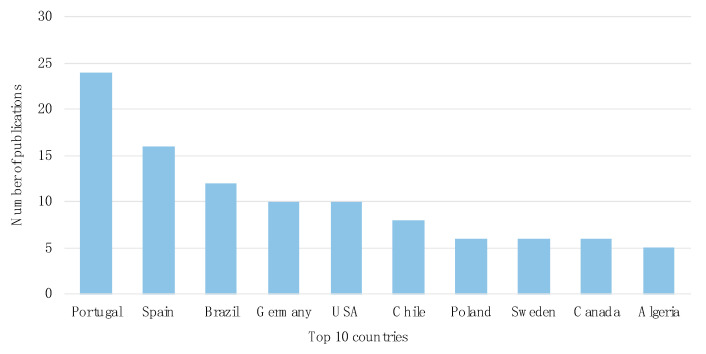
Distribution of publications among the top 10 publishing countries on cephalosporinases occurrence in wildlife.

A total of 46 countries published research describing cephalosporinases in wildlife over the last 18 years. Scientific production at the top 10 countries is presented in Figure 2, where it becomes evident that industrialized countries are the most productive, contributing to approximately 69% of total publications. Research is concentrated in European countries, especially Spain, Portugal, Germany, Poland, and Sweden. In addition, Brazil and Chile are prominent, as well as U.S.A and Canada, in South and North America, respectively. Characteristics transversal to European and North American countries, such as economic development that prompts substantial amounts of financial support to research activities, have direct impacts on academic productivity.

### 3.2. Host Taxonomy

**Figure 3:**
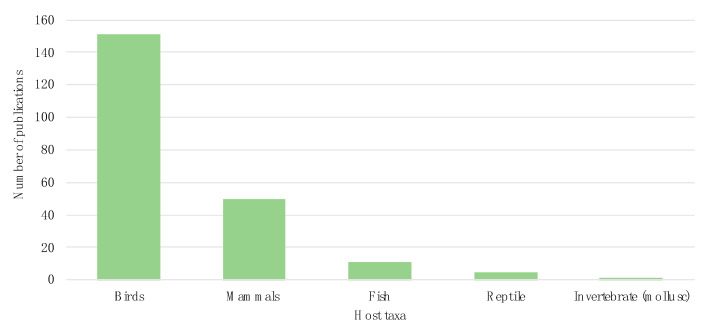
Number of cephalosporinases publications per wildlife host taxon.

There is a great diversity of wildlife species described as cephalosporinases hosts. In total, CB detection has been reported in 188 different species, with a clear taxonomic bias towards birds, as this is the taxon accounting for over half of the publications (151 publications, 69%) (Figure 3).

Birds have been widely under focus in AMR studies due to their long-range movements and exposure to anthropogenic sources, such as landfills and urban wastewater [19], acquiring and dispersing AMR bacteria within and between regions across long expanses of water, forestry, or desert on their migrations [20,21]. In fact, our results show that 48% of the publications related to birds focused on migratory species.

Among the birds category, different gull species are highlighted (Table 2). In fact, gulls are widely recognized as reservoirs of AMR, most likely due their common presence in urban and human waste areas, with underlying exposure to anthropogenic stressors [22,23].

Among mammals, almost half of the publications were on ungulates. This is most likely because of their wide abundance and distribution worldwide but also because some species may come in close contact with humans, not only because they establish a clear link between natural and humanized areas but also because they are game species in many countries, and access to fresh samples from hunter-harvested animals is thus easy. In Europe, hunting, whether as a cultural or wildlife management activity, provides indirectly a great opportunity to assess the risk of wild ungulates infectious diseases to public health. In 2015, according to the report of the United Nations Economic Commission for Europe [24], more than 23 tons of game meat were consumed and generated more than 321 million euros in Europe. These numbers show the large dimension of hunting in Europe, an activity that may also carry a risk for antimicrobial resistance dissemination due to the direct and indirect contact between humans and wildlife.

Among ungulates, wild boar was the species mostly reported to carry CB (Table 2); this species is also considered the most distributed mammal worldwide and today its expansion is a challenge for management, because in addition to the impacts on the ecological and financial levels, its expansion potentially impacts public health, since wild boar may harbour many zoonotic agents, including AMR bacteria.

Among the top 10 hosts (Table 2) are also peridomestic wildlife species, such as rats and gulls, as well as wolves, which prey on wild and domestic ungulates under extensive husbandry. These results suggest that AMR circulates within different trophic chains and that such species may be key elements of AMR dynamics in natural ecosystems, meaning that they could represent a major epidemiological link between domestic animals, humans, and wildlife. Furthermore, small anthropophilic prey species such as rodents could represent a bridge between human/domestic animals and their predators.

### 3.3. Cephalosporinase Diversity in Wildlife Hosts

**Figure 4:**
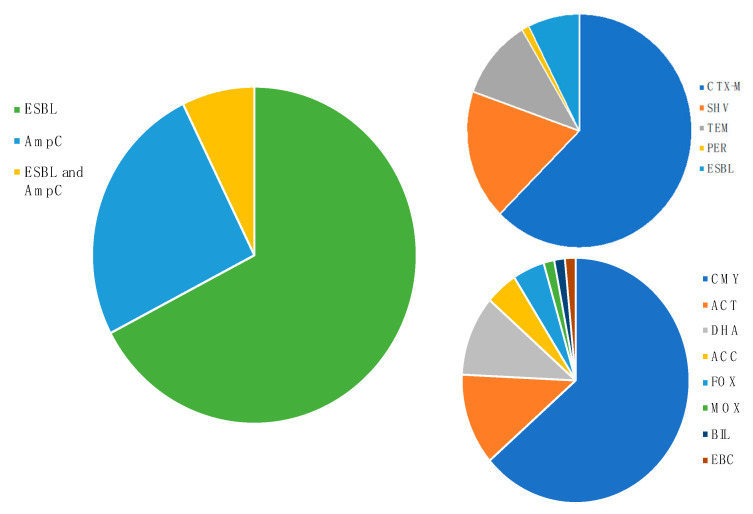
Proportion of publications by cephalosporinase (**left**), ESBL (**top right**) and AmpC beta-lactamase types (**bottom right**).

Data reported so far, suggest that wildlife may be a diversified reservoir of cephalosporinase types and other enzymes. ESBL stand out due to their presence in 96.6% of screened publications, more than double the number of reports describing AmpC producers (42.3%), while 9.4% report bacteria producing both types (Figure 4). The predominance of ESBLs is due to the molecular characteristics of their genes, since they are widely spread in mobile genetic elements, such as integrons and plasmids, unlike AmpCs that are mostly encoded in the chromosome [25]. Reports describing wildlife Enterobacteriaceae isolates with plasmids carrying resistance genes that encode ESBLs or AmpCs, are alarming situations, with public health, ecological, and economic consequences [26].

Five different types of ESBL have been described in wildlife, with 79.2% of the publications reporting CTX-M, 22.8% SHV, 14.1% TEM, 1.3% PER and 9.5% ESBL confirmed by phenotype without the description of the molecular type of gene (pESBL) (Figure 4). Interestingly, CTX-M, SHV and TEM are also the three main types of ESBL associated with therapeutic failures in patients with infections in hospital settings [27]. For CTX-M beta-lactamase, 23 variants were reported, highlighting CTX-M-1, -14, and -15, which together account for 83% of CTX-M reporting publications, the majority from *E. coli*. The CTX-M type ESBL is recognized as the most widespread ESBL around the world, being reported in all ecological niches, from humans (sick and healthy) to animals (livestock and pets) to the wider environment [28].

Despite being reported in fewer publications, AmpCs from wildlife showed a greater diversity of types than ESBL, eight different types being described (Figure 4) and with emphasis on CMY, that occurs in 28.2% of publications, followed by ACT (5.4%) and DHA (4.7%). CMY is the most widely disseminated AmpC globally and is described in several ecological niches, with emphasis on CMY-2, which is widely disseminated by plasmids [8]. CMY-2 was the most reported AmpC, with 63.3% of AmpC publications mentioning its detection in wildlife, which is in agreement with published data for the most widely disseminated AmpC status in other settings.

### 3.4. Wildlife and Cephalosporinase-Integrative Analyses

#### 3.4.1. Geographical Overview

**Figure 5:**
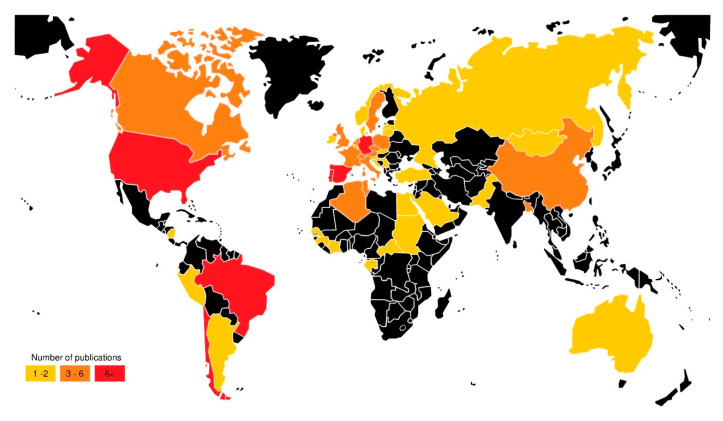
World map showing countries with publications reporting cephalosporinases in wildlife.

The geographic distribution of publications concerning the origins of CB in wildlife (Figure 5) shows the worldwide spread of cephalosporinases via this important component of ecosystems. The map also highlights a need for further investment in this animal niche. The underreporting in several countries, from Africa and Asia is most likely not due to an absence of CB in wildlife, but rather a lack of research [22].

**Figure 6:**
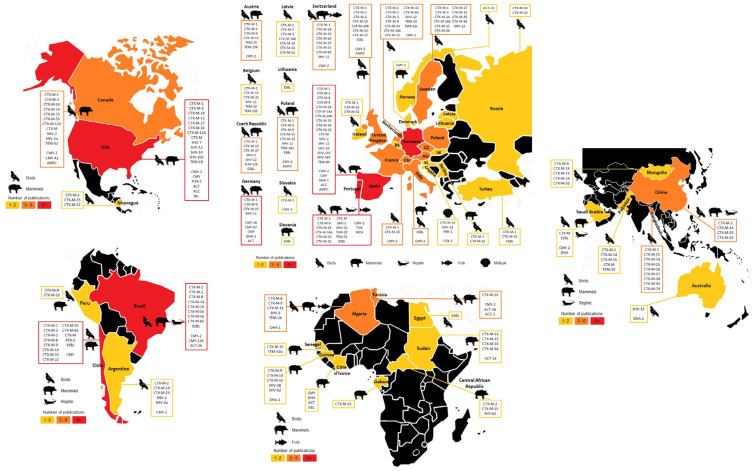
Maps of North and Central America (**top left**), South America (**bottom left**), Europe (**top center**), Africa (**bottom center**) and Asia and Oceania (**right**) showing the animal host and cephalosporinases subtypes described in the selected publications per country.

Different realities and characteristics stand out when analysing each continent separately. Europe, due to its public health policies, is the continent that reports more work on wild animals as reservoirs of cephalosporinases. The range of host taxa that carry cephalosporinases in Europe, includes birds, mammals, reptiles, fish and mollusks, which together totalize 106 different species as a reservoir of CB. There is a large variety of reported cephalosporinases (Figure 6), without a strikingly predominant profile, however CTX-M-15 is reported in 42 host species distributed in 18 countries, followed by CTX-M-1 from 42 animal species in 17 countries, CMY-2 described in 33 hosts from 11 countries, SHV-12 in 31 hosts from 10 countries and CTX-M-14 reported in 20 hosts from 8 countries (Table S1). According to the ECDC surveillance atlas of AMR, the prevalence of *E. coli* resistant to third generation cephalosporins in humans in the European Union, in 2018, ranged between 5 to 50%.

In North and Central America, only 3 countries (Figure 6) reported wildlife carrying CB, but with description of a high variety of cephalosporinases [25] and high-risk clones of *E. coli* (ST38, ST131, ST405 and ST648) from 29 hosts (birds and mammals). According to the U.S. Fish and Wildlife Service, 17 of the reported birds species are considered migratory, which is an important feature because it gives these birds the potential, as reservoirs of cephalosporinases, to disseminate CB and their genes in remote and urban areas, thus constituting a potential risk to human health [29].

An important point to highlight is that South America is worldwide recognized as an exporter of natural resources, including wildlife, which sets up a driver for the dissemination of zoonotic pathogens. A total of 33 wild animal species from 4 South-American countries (Argentina, Brazil, Chile, and Peru) are described as CB carriers (Figure 6). Among those, and besides CTX-M-15 (a global disseminated ESBL), CTX-M-2 and -8 that are typical enzymes arising from clinical settings from this continent, have been reported, and their association with high-risk clones (ST648) of *E. coli*, reported also in humans, livestock and environment in South America [28,30,31,32], bring concern.

Africa is a continent where wild animals have a great impact on human populations, since in many countries they are a public source of resources, whether for food, tourism, or cultural purposes [33]. The final set of selected publications cover only 9 countries from North and Central Africa (Figure 6), and they are still informative. CTX-M-15 was the predominant ESBL described in 18 species from 7 countries, including isolates from high-risk clones (ST38, ST131, ST405 and ST648) of *E. coli* carrying this gene. CTX-M-15 has been described in wildlife from 6 countries (Algeria, Senegal, Gabon, Central African Republic, Sudan and Egypt), where cephalosporinases are a resistance mechanism commonly associated with therapeutic failures in the hospital setting and community [34,35,36].

Similarly to Africa, in Asia, there is a need for more research in this field, since only 5 countries (Figure 6) have publications describing CB. It is a continent with cultural characteristics in which wild animals have a direct impact on humans and their health. A recent example is the SARS-CoV-2 pandemic, which has its probable origin in bats in China [37]. CTX-M-14 and -15 were the most described cephalosporinases (China, Pakistan, Bangladesh, and Mongolia), which are commonly associated with a high-risk clone of *E. coli* ST131 occurring in some *E. coli* isolates from Bangladesh and Mongolia; this clone has also been described in hospital settings in both countries [38,39].

Australia is the only country in Oceania (Figure 6) with a description of cephalosporinases in bacteria from wild animals, specifically in gulls (*Chroicocephalus novaehollandiae*) that harboured isolates of *K. pneumoniae* and *E. coli*, with SHV-12 and DHA-1, respectively. Both genes have been reported in the community and in hospitals, associated with intra-abdominal infections in the Asian-Pacific Region [40].

#### 3.4.2. Most Disseminated Cephalosporinases

**Figure 7:**
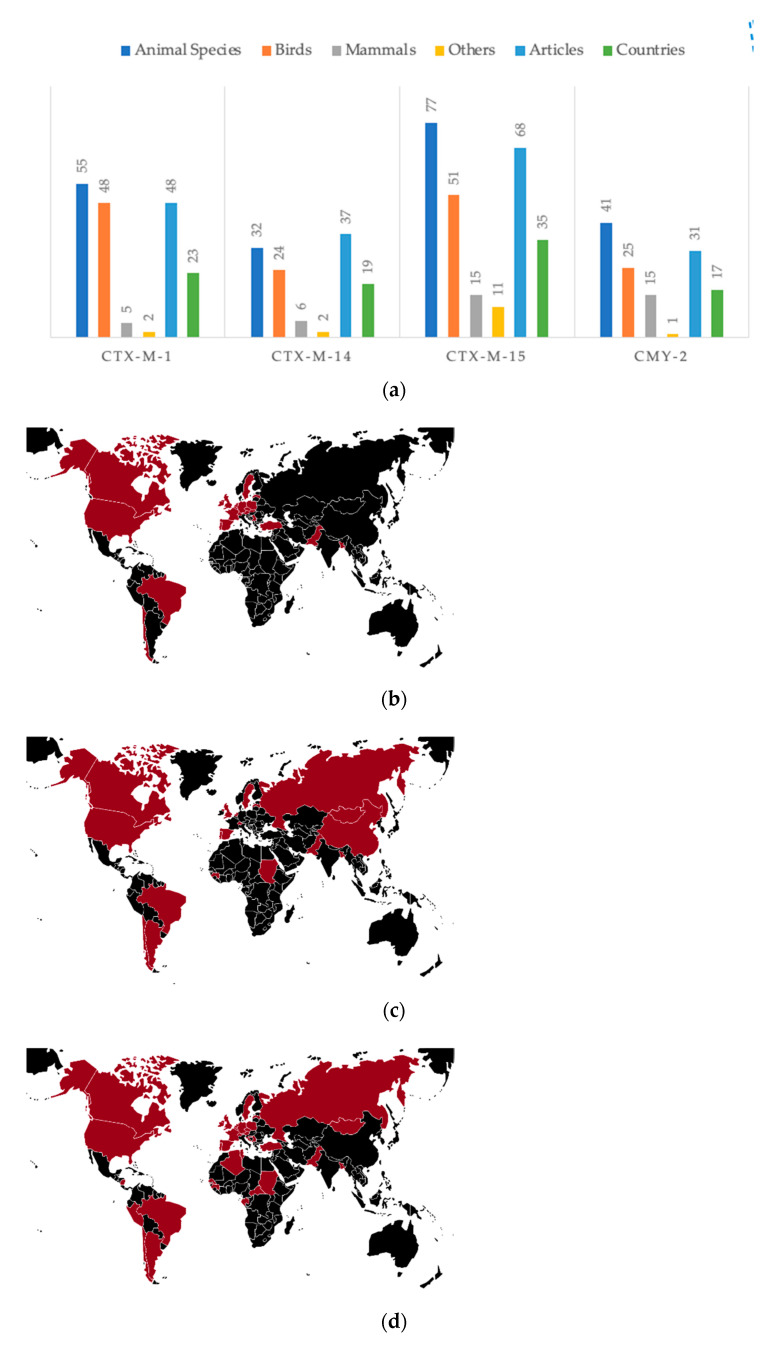

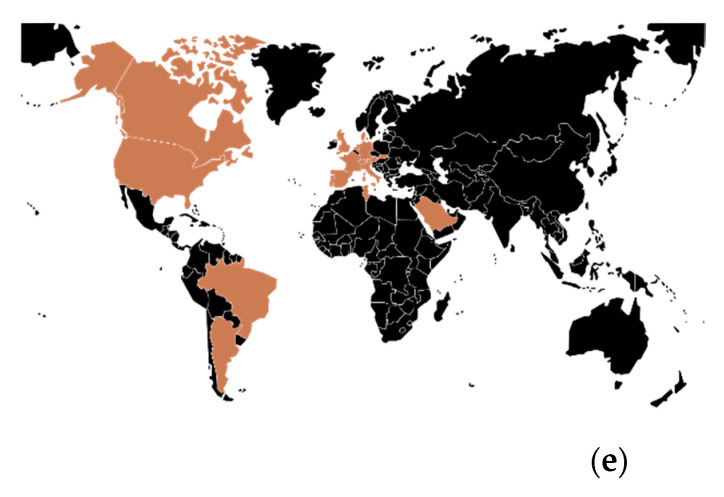
Summarizing data for the top 4 cephalosporinases reported in wildlife, including (**a**) animal species and taxa, publications and country numbers, and also the worldwide distribution of (**b**) CTX-M-1, (**c**) CTX-M-14, (**d**) CTX-M-15 (burgundy, ESBL beta-lactamases), and (**e**) CMY-2 (light brown, AmpC beta-lactamase) (others include fish, reptile, and invertebrate).

The diversity of cephalosporinases described in wildlife was high, but 4 of them were predominant, 3 ESBL (CTX-M-1, -14 and -15), and 1 AmpC (CMY-2). The CTX-M-15 was the most described with 68 publications, from 35 countries and present in 77 animal species. The beta-lactamase CTX-M-15 was detected in a variety of bacterial species or genera (*Aeromonas caviae*, *Aeromonas hydrophila*, *Citrobacter freundii*, *E. coli*, *Enterobacter cloacae*, *Enterobacter hormaechei*, *Enterobacter xiangfangensis*, *Escherichia fergusonii*, *Klebsiella pneumoniae*, *Klebsiella oxytoca*, *Morganella morganii*, *Proteus vulgaris*, *Providencia* spp.) [41] (Figure 7).

CTX-M-1 was the second most reported cephalosporinase (*n* = 55) from wildlife in 23 countries (Figure 7), although the publication incidence in Asia was low, while it was not reported in Africa. These last results might be explained by the taxon profile of CTX-M-1, its incidence in mammals is low (*n* = 5, 9.1%) and Africa publications is predominantly circumscribed to mammals (60%). Another feature is the low diversity of bacterial species (*C. freundii, E. coli, E. cloacae*) carrying this beta-lactamase. CTX-M-1 is an animal-associated ESBL but is also described in human bacterial infection in hospital settings [42,43].

The third most described cephalosporinase was CTX-M-14, reported in 37 publications from 19 countries, without strong distribution in Europe when compared with CTX-M-1 and -15, but frequently reported in Asia, where it was first described [43,44] (Figure 7). CTX-M-14 is an ESBL related with humans and animals worldwide. It is one of the most disseminated cephalosporinases, with high prevalence in some countries, such as Spain where it has been isolated from sick and healthy human individuals and is the most reported in wild animals [45].

CMY-2 is recognized worldwide as the most spread AmpC cephalosporinase, associated with the incidence of Enterobacteriales in human infection, and this is not different in wild animals, with 31 publications from 17 countries, carried by 41 animal species; CMY-2 is reportedly detected from *C. freundii*, *E. coli*, *E. cloacae*, *K. pneumoniae*, *Proteus mirabilis* and *S. enterica* serovar Heidelberg, with geographic distribution across Europe, North America, and South America [46] (Figure 7).

#### 3.4.3. Cephalosporinases and Clonality

Bacterial clones are classified according to their phylogenetic features, with some clones, the high-risk clones, showing adaptative capacity to colonize, spread, persist, and increase virulence, posing a threat to human and animal health [47]. Wild animals may harbour cephalosporinases-producing bacteria, as well as high-risk clone bacteria, namely clones of *E. coli*, with potential impacts on public health. Six high-risk clones of *E. coli* have been described (ST131, ST38, ST648, ST405, ST155, and ST69), with highlight to ST131, the most widespread high-risk clone of *E. coli* responsible for infection outbreaks worldwide [47].

**Figure 8:**
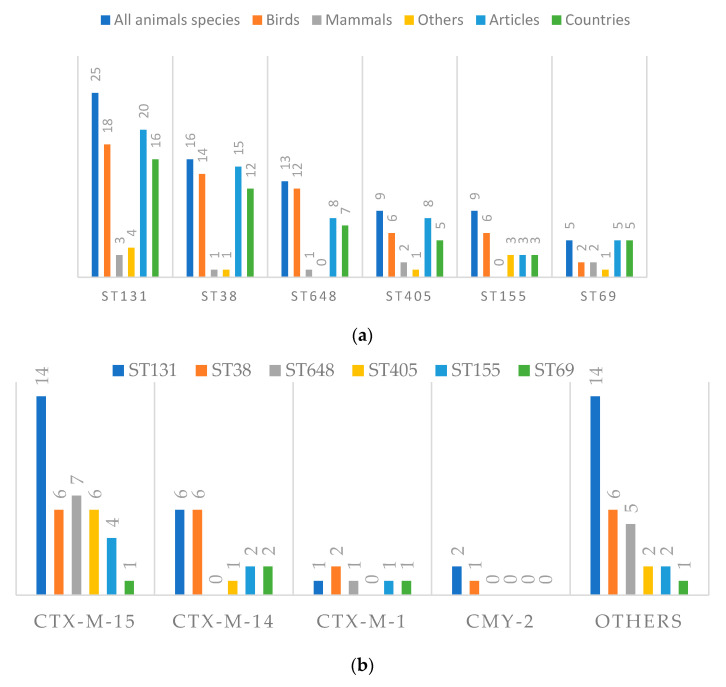

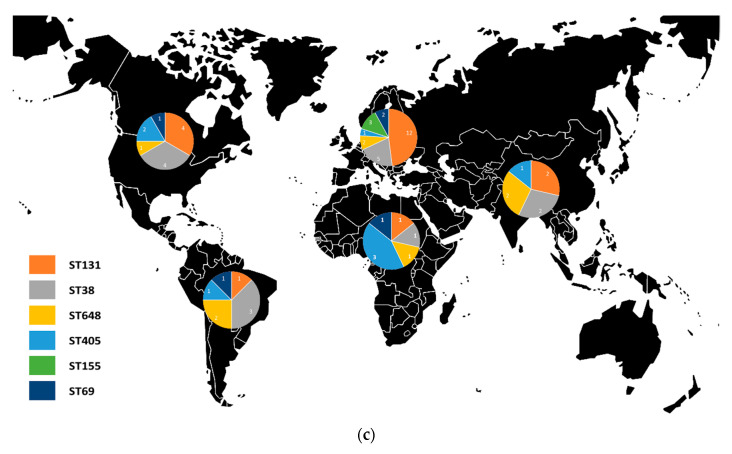
Details of high-risk clones of *E. coli* associated with cephalosporinases in wild animals, including (**a**) animals species and taxa, publications and country numbers, (**b**) their co-occurrence with the top 4 reported cephalosporinases and (**c**) distribution per continent.

The combination of antibiotic resistance and virulence of pathogenic bacteria is a serious threat to human health and this combination is present in wild animals. Figure 8 shows the relation between the most reported cephalosporinases that are highly prevalent in human infections too, and the high-risk clones of *E. coli*, responsible for worldwide outbreaks in hospital settings and in the community [47]. CTX-M-15 was the most reported cephalosporinase in these important clones, being described in 12.8% of the publications from 16 countries, present in all high-risk clones of *E. coli* that strongly relate to ST131 that circulate in the human sphere worldwide [47]. Clones of ST131/CTX-M-15 in wild animals have been described in 12 countries from Africa, Asia, North America, South America, and Europe, highlighting its spreading potential. The other reported high-risk clones of *E. coli* have been described in these five continents too, except for ST155, which is present only in Europe.

Well adapted to humans, the extent to which CTX-M-15 producing ST131 *E. coli* clone is transmitted among different ecological niches remains unknown but ST131 isolates from diverse ecological sources exhibited broad genetic commonality, providing strong evidence that this pandemic clone spreads between different ecological niches at the human-environment-wildlife interface [48]. In addition, wild fish in the Mediterranean Sea show plasmid-mediated oxyimino-cephalosporin-resistant *E. coli*, including the pandemic clone B2-O25b:H4-ST131 [49].

**Figure 9:**
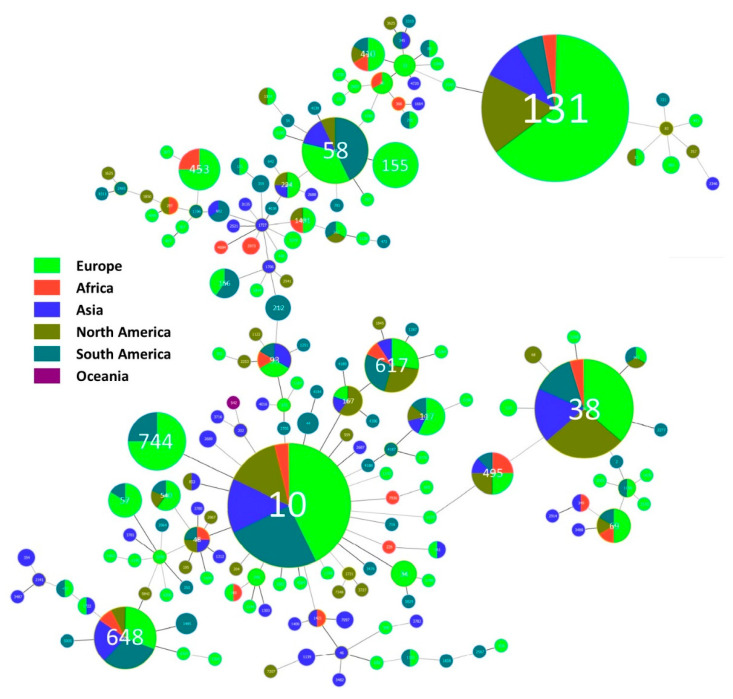
MLST-based minimal spanning tree of 423 ESBL- and AmpC-producing *E. coli* in wild animals from Europe, Africa, Asia, North America, South America, and Oceania. Node sizes reflect the number of isolates with specific MLST profile. Numbers within the nodes indicate the ST. Node colours refer to the continent origin of isolates with each specific ST. Nodes differing by one, two, or three loci are linked by dark lines. MLST, multilocus sequence typing, ST, sequence type.

A minimal spanning tree of 423 cephalosporinase-producing *E. coli* (Figure 9) highlights the clonal diversity of *E. coli* from wild animals, but with few non-high-risk clones, such as ST58, ST744, ST617, ST495, and ST453, with a high number of reports. Despite these clones not being high-risk clones, all of them have been described as a cause of infection and/or dissemination in human or non-wild animals [50,51,52]. The clonal diversity of cephalosporinases-producing *E. coli* from wild animals may reflect the different types and intensities of anthropogenic pressure. Since the typing features of each strain can be related with pressure environment type, this pressure can also impact the mobile horizontal transfer spread of cephalosporinases, mainly ESBL. AmpC can have intrinsic resistance in some bacteria species, and it is very important to understand the clonal circulation to crosslink the ecosystem compartments [53,54].

This whole scenario shows that wildlife can be a reservoir of AMR, but also a mirror of how the human activities, mainly those with high antimicrobial usage level such as human clinical settings and livestock, affect the whole ecosystem. Antimicrobial exposure, either indirectly (shared habitat, contaminated water) or directly (recue centres, veterinary hospitals), is the main AMR spread driver [22].

The pandemic spread of certain ESBL-*E. coli* lineages showing high diversity of extended-spectrum β-lactamases from wildlife helps to understand transmission dynamics [55], supporting the concept that wildlife is a good sentinel for resistance determinants, underlining the importance of the One Health approach [56].

## 4. Conclusions

Dissemination of CB, including high-risk clones, in healthcare and community settings, as well as among wildlife animals, indicates that CB have high capacity to jump from anthropogenic to natural ecosystems and vice-versa, creating parallel reservoirs that contribute to the spread and evolution of these enzymes. It is not easy to determine the routes or the drivers that have influenced AMR spread to the environment, namely to wildlife animals, but is clear that wildlife is an important part of the AMR ecosystem, as a vehicle and maybe as a reservoir of AMR determinants to human and livestock animals under extensive husbandry, due to the intrinsic connection between these niches. Targeting interventions to address dissemination of AMR and zoonotic pathogens such as *Enterobacteriaceae* relies on determining the vehicles for such infections. Surveillance under the One Health approach is thus key to understand the extent and routes of AMR propagation, as well as to comprehend the driving forces of the interaction between AMR and wildlife.

## Figures and Tables

**Table 1:** Number of publications reporting cephalosporinases in wildlife per continent.

Continent	Number of Publications
Europe	81
South America	21
North America	16
Africa	15
Asia	13
Oceania	2
Central America	1

**Table 2:** Top 10 of wildlife host species (mammals and birds) with reported cephalosporinases occurrence.

		Species	Number of Publications
Mammals	Ungulates	Wild boar (*Sus scrofa*)	9
		Roe deer (*Capreolus capreolus*)	6
		Red deer (*Cervus elaphus*)	4
		Mouflon (*Ovis orientalis musimon*)	3
	Small mammals	Brown rat (*Rattus norvegicus*)	5
		Hedgehog (*Erinaceus europaeus*)	3
		Black rat (*Rattus rattus*)	3
	Carnivores	Red fox (*Vulpes vulpes*)	5
		Iberian wolf (*Canis lupus signatus*)	2
		Badger (*Meles meles*)	2
Birds	Gulls	Herring gull (*Larus argentatus*)	15
		Yellow-legged gull (*Larus michahellis*)	12
		Lesser black-backed gull (*Larus fuscus*)	6
		Black-backed gull (*Chroicocephalus ridibundus*)	6
	Birds of prey	Black kites (*Milvus migrans*)	8
		Common buzzard (*Buteo buteo*)	7
	Pigeons	Pigeon (*Columba livia*)	8
	Ducks	Duck (*Anas platyrhynchos*)	6
	Rooks	Rooks (*Corvus frugilegus*)	6

## Supplementary Materials

The following are available online at https://www.mdpi.com/article/10.3390/ani11061765/s1, Figure S1: Flowchart of the literature search, Table S1: Description of number of bacterial origins, publication data and host taxonomy information of cephalosporinases reported in wild animals; List of revised papers.
