# Supplementary material for: Emergence and Spread of Cephalosporinases in Wildlife: A Review
Source: Animals (Basel). 2021 Jun 12;11(6):1765. doi: 10.3390/ani11061765 (PMC8231518; doi:10.3390/ani11061765)
Supplement: Supplementary file 1 [file animals-11-01765-s001.zip › animals-1245430-supplementary.pdf]

**SUPPLEMENTARY MATERIAL**

Table S1 – Description of number of bacterial origins, publication data and host taxonomy information of cephalosporinases reported in wild animals

| Beta-lactamase | Number of Paper | Bacteria species (with Sequence Typing) | Geographical Location | | Host Taxonomy | | | Reference |
|---|---|---|---|---|---|---|---|---|
| | | | Continents | Countries | Birds | Mammals | Others | |
| CTX-M-1 | 48 | *Citrobacter freundii* *E. coli* (ST10, ST1122, ST1140, ST1142, ST1143, ST1144, ST115, ST117, ST1199, ST12, ST1204, ST1284, ST1304, ST131, ST1431, ST1462, ST155, ST156, ST1586, ST162, ST1640, ST167, ST1670, ST1683, ST1730, ST1968, ST205, ST2198, ST2199, ST224, ST23, ST2607, ST34, ST3625, ST373, ST3778, ST3781, ST38, ST398, ST4306, ST4720, ST540, ST58, ST648, ST681, ST69, ST744, ST744, ST847, ST857, ST88, ST90) *Enterobacter cloacae* | Asia Europe North America South America | Austria, Bangladesh, Belgium, Brazil, Canada, Chile, Czech Republic, England, France, Germany, Ireland, Latvia, Netherlands, Pakistan, Poland, Portugal, Serbia, Slovakia, Spain, Sweden, Switzerland, Turkey, USA | *Accipiter gentilis, Accipiter nisus, Aegypius monachus, Alopochen aegyptiaca, Anas platyrhynchos, Anas strepera, Ardea cinereal, Bubo bubo, Buteo buteo, Buteo jamaicensis, Buteo lineatus, Buteo platypterus, Chroicocephalus ridibundus, Ciconia Ciconia, Circus pygargus, Columba livia, Corvus brachyrhynchos, Corvus corax, Corvus corone, Corvus frugilegus, Corvus splendens, Croicocephalus ridibundus, Cygnus olor, Dendrocopos major, Falco sparverius, Falco tinnunculus, Fulica atra, Gyps fulvus, Hieraaetus fasciatus, Hieraaetus pennatus, Larus argentatus, Larus cachinnans, Larus canus, Larus fuscus, Larus marinus, Larus michahellis, Leucophaeus pipixcan, Megascops asio, Megascops choliba, Melopsittacus undulates, Milvus migrans, Milvus milvus, Morus bassanus, Spheniscus magellanicus, Strix aluco, Strix varia, Tyto alba, Vanellus vanellus* | *Canis lupus signatus, Capreolus capreolus, Cervus elaphus, Rattus norvegicus, Sus scrofa* | *Rutilus rutilus, Coregonus lavaretus* | (Flavia Zendri et al., 2020, Zurfluh et al., 2019, Ngaiganam et al., 2019, Desvars-Larrive et al., 2019, Mughini-Gras et al., 2019, Hathcock et al., 2019, Jamborova et al., 2018a, Batalha de Jesus et al., 2019, Velhner et al., 2018, Hessman et al., 2018, Atterby et al., 2017, Mohsin et al., 2017, Wasyl et al., 2018, Mateus-Vargas et al., 2017, Sellera et al., 2017, Mathys et al., 2017a, Timonin et al., 2017, Cristóvão et al., 2017, Vergara et al., 2017, Yılmaz and Dolar, 2017, Alcalá et al., 2016, Monteiro et al., 2016, Alonso et al., 2016, Schaufler et al., 2016, Stedt et al., 2015, Hasan et al., 2015, Bonnedahl et al., 2015, Rouffaer et al., 2014, Abgottspon et al., 2014, Loncaric et al., 2014, Veldman et al., 2013, Hernandez et al., 2013, Guenther et al., 2013b, Kmet et al., 2013, Guenther et al., 2013a, Stephan and Hächler, 2012, Simões et al., 2012, Wallensten et al., 2011, Literak et al., 2010a, Pinto et al., 2010, Literak et al., 2010b, Simões et al., 2010, Radhouani et al., 2010, Poeta et al., 2009, Bonnedahl et al., 2009, Dolejska et al., 2009, Poeta et al., 2008, Costa et al., 2006) |
| CTX-M-2 | 8 | *E. coli* (ST10, ST101, ST1011, ST1158, ST117, ST2277, ST2485, ST359, ST38, ST4187, ST4188, ST4190, ST457, ST57, ST58, ST617, ST642, ST648, ST69, ST93), *Kluyvera ascorbata* | Africa, Central America, Europe, South America | Argentina, Brazil, Central African Republic, Chile, England, Latvia, Netherlands, Nicaragua | *Amazona aestiva, Caracara plancus, Columba livia, Coragyps atratus, Larus argentatus, Larus dominicanus, Larus fuscus, Leucophaeus pipixcan* | *Nasua nasua, Gorilla gorilla gorilla* | - | (de Carvalho et al., 2020, Marcos Paulo Vieira et al., 2019, Batalha de Jesus et al., 2019, Hasan et al., 2016, Liakopoulos et al., 2016, Stedt et al., 2015, Báez et al., 2015, Janatova et al., 2014) |

| | | | | | | | | |
|---|---|---|---|---|---|---|---|---|
| CTX-M-3 | 14 | *Aeromonas hydrophila, Citrobacter spp., E. coli* (ST1079, ST350, ST744), *Klebsiella oxytoca, Klebsiella spp.* | Asia, Europe, North America, South America | Austria, Canada, Chile, China, England, Latvia, Netherlands, Poland, Spain, Sweden, USA | *Alopochen aegyptiaca, Anas platyrhynchos, Bucephala clangula, Corvus frugilegus, Larus argentatus, Larus fuscus, Larus michahellis, Leucophaeus pipixcan, Uria aalge* | *Myotis daubentonii, Plecotus auratus, Rattus norvegicus, Atelerix algirus* | *Chelydra serpentine* | (Nowakiewicz et al., 2020, Desvars-Larrive et al., 2019, Darwich et al., 2019, Mughini-Gras et al., 2019, Zou et al., 2019, Hessman et al., 2018, Mathys et al., 2017a, Deng et al., 2016, Stedt et al., 2015, Bonnedahl et al., 2015, Báez et al., 2015, Loncaric et al., 2014, Veldman et al., 2013, Hernandez et al., 2013) |
| CTX-M-8 | 8 | *Citrobacter freundii, E. coli* (ST10, ST212, ST2705, ST345, ST4138, ST48, ST56, ST58, ST602, ST648), *Enterobacter cloacae* | Africa, Europe, South America | Algeria, Brazil, Chile, Peru, Spain | *Amazona aestiva, Bubo magellanicus, Caracara plancus, Columba livia, Coragyps atratus, Larus michahellis, Mergus octosetaceus, Strix rufipes* | *Leopardus pardalis* | - | (Fuentes-Castillo et al., Furlan et al., 2020, Marcos Paulo Vieira et al., 2019, Fuentes-Castillo et al., 2019, Batalha de Jesus et al., 2019, Brahmi et al., 2018, Borges et al., 2017, Stedt et al., 2015) |
| CTX-M-9 | 11 | *E. coli* (ST13, ST131, ST167, ST2346, ST38, ST502, ST58, ST648), *K. pneumoniae, Salmonella enterica* serovar Agona | Africa, Asia, Europe, South America | Algeria, Austria, Chile, Germany, Guinea, Mongolia, Netherlands, Poland, Portugal, Spain | *Anthropoides virgo, Aegypius monachus, Larus argentatus, Larus audouinii, Larus cachinnans, Larus fuscus, Larus michahellis, Leucophaeus pipixcan, Milvus migrans* | *Canis lupus signatus, Rattus norvegicus, Rattus rattus* | *Pagellus acarne* | (Desvars-Larrive et al., 2019, Brahmi et al., 2018, Schaufler et al., 2018, Stedt et al., 2015, Bonnedahl et al., 2015, Antilles et al., 2015, Guenther et al., 2013a, Simões et al., 2012, Literak et al., 2010a, Guenther et al., 2010b, Simões et al., 2010) |
| CTX-M-11 | 1 | *E. coli* (ST1312) | Asia | Bangladesh | *Aythya fuligula* | - | - | (Hasan et al., 2012) |
| CTX-M-14 | 31 | *E. coli* (ST1196, ST10, ST117, ST131, ST1485, ST155, ST167, ST2253, ST2973, ST38, ST405, ST448, ST453, ST58, ST609, ST69, ST744, ST7936, ST88, ST896), *K. pneumoniae, Proteus mirabilis* | Africa, Asia, Europe, North America, South America | Argentina, Bangladesh, Brazil, Canada, Chile, China, England, Guinea, Mongolia, Netherlands, Pakistan, Portugal, Russia, Spain, Sudan, Sweden, Switzerland, USA | *Aegypius monachus, Anas platyrhynchos, Aythya fuligula, Chroicocephalus brunnicephalus, Chroicocephalus ridibundus, Coragyps atratus, Corvus brachyrhynchos, Corvus corax, Corvus splendens, Cygnus olor, Larus argentatus, Larus canus, Larus dominicanus, Larus fuscus, Larus marinus, Larus michahellis, Leucophaeus pipixcan, Pastor roseus, Sylvia atricapilla, Tringa tetanus, Vultur gryphus Linnaeus* | *Vulpes zerda, Rattus rattus, Canis lupus signatus* | *Rutilus rutilus, Coregonus lavaretus* | (Flavia Zendri et al., 2020, Fuentes-Castillo et al., 2020, de Carvalho et al., 2020, Feng et al., 2019, Mughini-Gras et al., 2019, Ahlstrom et al., 2019, Zou et al., 2019, Jamborova et al., 2018a, Schaufler et al., 2018, Hessman et al., 2018, Atterby et al., 2017, Guenther et al., 2017, Raza et al., 2017, Cristóvão et al., 2017, Vergara et al., 2017, Liakopoulos et al., 2016, Stedt et al., 2015, Hasan et al., 2015, Bonnedahl et al., 2015, Hasan et al., 2014, Gonçalves et al., 2014, Bonnedahl et al., 2014, Abgottspon et al., 2014, Veldman et al., 2013, Hernandez et al., 2013, Silva et al., 2011, Wallensten et al., 2011, Bonnedahl et al., 2010, Hernandez et al., 2010, Costa et al., 2008, Costa et al., 2006) |
| CTX-M-14a | 7 | *E. coli* (ST2001, ST117, ST131, ST877) | Europe | Portugal, Spain | *Milvus migrans, Tyto alba* | *Neovison vison, Lynx pardinus* | - | (Alonso et al., 2017a, Cristóvão et al., 2017, Alcalá et al., 2016, |

| | | | | | | | | |
|---|---|---|---|---|---|---|---|---|
| | | | | | | | | Monteiro et al., 2016, Gonçalves et al., 2012, Poeta et al., 2008) |
| CTX-M-14b | 1 | Not specified | Europe | England, Latvia, Netherlands, Spain, Sweden | *Larus michahellis, Larus argentatus, Larus fuscus* | - | - | (Stedt et al., 2015) |
| CTX-M-15 | 68 | *Aeromonas caviae, Aeromonas hydrophila, Citrobacter freundii* (ST265), *E. coli* (ST10, ST1139, ST1139, ST1193, ST12, ST120, ST1251, ST1303, ST131, ST1408, ST1421, ST1431, ST1485, ST155, ST156, ST165, ST1664, ST1706, ST1722, ST1727, ST1788, ST2, ST202, ST205, ST2141, ST224, ST226, ST23, ST2521, ST2687, ST2688, ST2689, ST2914, ST297, ST305, ST3135, ST3268, ST34, ST345, ST3476, ST3479, ST3482, ST3487, ST3488, ST349, ST350, ST354, ST361, ST3716, ST3780, ST3782, ST38, ST4016, ST405, ST410, ST4184, ST4185, ST4186, ST4189, ST44, ST46, ST4684, ST472, ST48, ST491, ST5005, ST540, ST559, ST58, ST602, ST617, ST636, ST648, ST69, ST7097, ST716, ST721, ST744, ST746, ST767, ST853, ST90, ST93), *Enterobacter cloacae* (ST279), *Enterobacter hormaechei* (ST114), *Enterobacter xiangfangensis* (ST114), *Escherichia fergusonii, K. pneumoniae* (ST1211, ST14 , ST219, ST307, ST377, ST39, ST405, ST584), *Klebsiella oxytoca, Morganella morganii, Proteus vulgaris, Providencia spp.*, Only ARG | Africa, Asia, Central America, Europe, North America, South America | Algeria, Argentina, Austria, Bangladesh, Belgium, Brazil, Canada, Central African Republic, Chile, Croatia, Czech Republic, England, France, Gabon, Germany, Guinea, Ireland, Latvia, Mongolia, Netherlands, Nicaragua, Pakistan, Peru, Poland, Portugal, Russia, Senegal, Serbia, Spain, Sudan, Sweden, Switzerland, Tunisia, Turkey, USA | *Accipiter nisus, Anas clypeata, Anas crecca, Anas penelop, Anas platyrhynchos, Anastomus oscitans, Anser albifrons, Anser fabalis, Ardea cinereal, Bubo bubo, Bubulcus ibis, Buteo buteo, Caracara plancus, Carduelis carduelis, Carduelis chloris, Chroicocephalus brunnicephalus, Chroicocephalus ridibundus, Ciconia Ciconia, Columba livia, Coragyps atratus, Corvus brachyrhynchos, Corvus frugilegus, Corvus splendens, Croicocephalus ridibundus, Cygnus olor, Falco naumanni, Fulica atra, Larus argentatus, Larus cachinnans, Larus canus, Larus delawarensis, Larus dominicanus, Larus fuscus, Larus glaucescens, Larus marinus, Larus michahellis, Leucophaeus pipixcan, Megascops choliba, Melopsittacus undulates, Merops apiaster, Milvus migrans, Morus bassanus, Netta rufina, Pastor roseus, Phalacrocorax carbo, Rupornis magnirostris, Serinus serinus, Strix aluco, Sylvia melanocephala, Taeniopygia guttata, Turdus merula* | *Desmodus rotundus, Epomops franqueti, Erinaceus europaeus, Gorilla gorilla gorilla, Macaca Sylvanus, Megaloglossus woermanni, Myotis daubentoniid, Nasua nasua, Ovis orientalis musimon, Plecotus auratus, Rattus norvegicus, Rattus rattus, Sus scrofa, Tapirus terrestris, Vulpes zerda* | *Abramis brama, Bothrops alternatus, Chelonia mydas, Coregonus lavaretus, Mytilu galloprovincialis, Pagellus acarne, Perca fluviatilis, Rutilus rutilus, Salmo trutta, Sardina pilchardus, Sarpa salpa* | (Flavia Zendri et al., 2020, de Carvalho et al., 2020, Nowakiewicz et al., 2020, Mbehang Nguema et al., 2020, Ben Yahia et al., 2020, Feng et al., 2019, Zurfluh et al., 2019, Ngaiganam et al., 2019, Desvars-Larrive et al., 2019, Darwich et al., 2019, Mughini-Gras et al., 2019, Goldberg et al., 2019, Ahlstrom et al., 2019, Mairi et al., 2019, de Carvalho et al., 2018, Jamborova et al., 2018b, Batalha de Jesus et al., 2019, Benavides et al., 2018, Oteo et al., 2018, Bouaziz et al., 2018, Ben Yahia et al., 2018, Brahmi et al., 2018, Schaufler et al., 2018, Hessman et al., 2018, Atterby et al., 2017, Mohsin et al., 2017, Taous Bachiri et al., 2018, Wasyl et al., 2018, Mathys et al., 2017b, Guenther et al., 2017, Mathys et al., 2017a, Raza et al., 2017, Bachiri et al., 2017, Vergara et al., 2017, Yılmaz and Dolar, 2017, Hasan et al., 2016, Liakopoulos et al., 2016, Parker et al., 2016, Jones-Dias et al., 2016, Loncaric et al., 2016, Atterby et al., 2016, Schaufler et al., 2016, Stedt et al., 2015, Hasan et al., 2015, Bonnedahl et al., 2015, Báez et al., 2015, Rashid et al., 2015, Rouffaer et al., 2014, Hasan et al., 2014, Janatova et al., 2014, Bonnedahl et al., 2014, Abgottspon et al., 2014, Loncaric et al., 2014, Veldman et al., 2013, Hernandez et al., 2013, Maravić et al., 2013, Zurfluh et al., 2013, Klimes et al., 2013, Hasan et al., 2012, Poirel et al., 2012, Literak et al., 2010a, Guenther et al., 2010a, Bonnedahl et al., 2010, Hernandez et al., 2010, |

| | | | | | | | | |
|---|---|---|---|---|---|---|---|---|
| | | | | | | | | Simões et al., 2010, Ivan Literak et al., 2009, Bonnedahl et al., 2009, Dolejska et al., 2009) |
| CTX-M-16 | 2 | *Serratia marcensis, Raoultella terrigena* | Asia, Europe | Bangladesh, Spain | *Accipiter nisus, Corvus splendens* | - | - | (Darwich et al., 2019, Hasan et al., 2015) |
| CTX-M-17 | 1 | *Enterobacter cloacae* | Asia | Bangladesh | *Corvus splendens* | - | - | (Hasan et al., 2015) |
| CTX-M-18 | 1 | *Citrobacter freundii* | Asia | Bangladesh | *Corvus splendens* | - | - | (Hasan et al., 2015) |
| CTX-M-22 | 1 | *E. coli* (ST10, ST131, ST3476, ST359, ST4187, ST4188, ST540, ST744) | South America | Chile | *Leucophaeus pipixcan* | - | - | (Báez et al., 2015) |
| CTX-M-24 | 3 | *E. coli* (ST38) | Asia, Europe | Mongolia, Sweden, Switzerland | *Larus argentatus, Milvus migrans* | - | *Rutilus rutilus* | (Guenther et al., 2017, Stedt et al., 2015, Abgottspon et al., 2014) |
| CTX-M-27 | 7 | *E. coli* (ST131, ST2245, ST38, ST405) | Europe, North America | Czech Republic, England, Sweden, Switzerland, USA | *Corvus brachyrhynchos, Corvus frugilegus, Croicocephalus ridibundus, Larus argentatus, Larus canus, Larus fuscus, Larus marinus, Phalacrocorax carbo* | - | *Centrarchidae, Coregonus lavaretus, Rutilus rutilus, Salmo trutta* | (Flavia Zendri et al., 2020, Jamborova et al., 2018b, Atterby et al., 2017, Stedt et al., 2015, Bonnedahl et al., 2014, Abgottspon et al., 2014, Zurfluh et al., 2013) |
| CTX-M-32 | 13 | *E. coli* (ST10, ST131, ST1845, ST38, ST681, ST853) | Central America, Europe, North America | Belgium, Canada, Ireland, Latvia, Netherlands, Nicaragua, Poland, Portugal, Spain, Sweden, USA | *Anas platyrhynchos, Bubulcus ibis, Buteo buteo, Columba livia, Corvus brachyrhynchos, Corvus corax, Croicocephalus ridibundus, Cygnus atratus, Falco tinnunculus, Larus argentatus, Larus cachinnans, Larus canus, Larus delawarensis, Larus fuscus, Larus marinus, Larus michahellis, Tringa totanus* | *Vulpes vulpes* | - | (Díaz-Jiménez et al., 2020, Mughini-Gras et al., 2019, Jamborova et al., 2018a, Atterby et al., 2017, Hasan et al., 2016, Monteiro et al., 2016, Stedt et al., 2015, Rouffaer et al., 2014, Veldman et al., 2013, Poirel et al., 2012, Simões et al., 2010, Radhouani et al., 2010, Poeta et al., 2008) |
| CTX-M-55 | 13 | *E. coli* (ST117, ST1193, ST155, ST162, ST165, ST212, ST2973, ST366, ST453, ST48, ST58, ST744, ST90, ST93) | Africa, Asia, Europe, North America, South America | Bangladesh, Brazil, Canada, Chile, China, Latvia, Mongolia, Sudan, Sweden, Switzerland | *Aegypius monachus, Anas platyrhynchos, Coragyps atratus, Corvus corone, Corvus frugilegus, Corvus splendens, Croicocephalus ridibundus, Larus argentatus, Larus canus, Larus marinus, Leucophaeus pipixcan, Vultur gryphus Linnaeus* | *Ailuropoda melanoleuca, Nasua nasua, Vulpes zerda* | - | (Fuentes-Castillo et al., 2020, de Carvalho et al., 2020, Feng et al., 2019, Zurfluh et al., 2019, Söderlund et al., 2019, Zhou et al., 2018, Hessman et al., 2018, Atterby et al., 2017, Stedt et al., 2015, Hasan et al., 2015, Bonnedahl et al., 2015, Ho et al., 2013, Guenther et al., 2013a) |
| CTX-M-56 | 1 | *E. coli* (ST212) | South America | Brazil | *Asio clamator* | - | - | (de Carvalho et al., 2020) |
| CTX-M-64 | 1 | *E. coli* (ST48) | Africa | Sudan | - | *Vulpes zerda* | - | (Feng et al., 2019) |
| CTX-M-65 | 7 | *E. coli* (ST156, ST58, ST602), *Salmonella enterica* serovar Infantis (ST32) | Asia, Europe, South America | Brazil, Chile, China, Netherlands, Sweden, Switzerland | *Anas platyrhynchos, Bubo magellanicus, Larus argentatus, Larus fuscus, Turdus merula, Vultur gryphus Linnaeus* | *Myrmecophaga tridactyla* | - | (Furlan et al., 2020, Fuentes-Castillo et al., 2020, Zurfluh et al., 2019, Fuentes-Castillo et al., 2019, Zou et al., 2019, Hessman et al., 2018, Stedt et al., 2015) |
| CTX-M-79 | 1 | *E. coli* | Asia | Bangladesh | *Corvus splendens* | - | - | (Hasan et al., 2015) |

| | | | | | | | | |
|---|---|---|---|---|---|---|---|---|
| CTX-M-124 | 2 | *E. coli* (ST5842, ST648) | North America | Canada, USA | *Corvus brachyrhynchos, Corvus corax, Larus delawarensis* | - | - | (Jamborova et al., 2018a, Poirel et al., 2012) |
| CTX-M | 10 | *Aeromonas veronii, Citrobacter freundii, E. coli* (ST131, ST2541, ST297, ST68), *Enterobacter cloacae, Enterobacter spp., K. pneumoniae, Klebsiella oxytoca, Serratia fonticola, Stenotrophomonas maltophilia, Stenotrophomonas nitritireducens,* Only ARG | Asia, Europe, North America, South America | Canada, Chile, Pakistan, Portugal, Saudi Arabia, Slovenia, Spain, USA | *Accipiter nisus, Anas platyrhynchos, Aquila chrysaetos, Aquila fasciata, Bubo bubo, Bubulcus ibis, Carduelis yemenensis, Chroicocephalus ridibundus, Ciconia Ciconia, Circaetus gallicus, Clamator glandarius, Columba livia, Coragyps atratus, Corvus brachyrhynchos, Corvus corax, Dendrocopos major, Falco tinnunculus, Fulica atra, Gyps fulvus, Hieraaetus pennatus, Larus argentatus, Larus fuscus, Milvus migrans, Psittacus erithacus, Streptopelia turtur, Strix aluco, Tyto alba* | *Capreolus capreolus, Leopardus guigna, Papio hamadryas, Rattus norvegicus, Rattus rattus* | - | (Sacristán et al., 2020, Bueno et al., 2020, Sen et al., 2019, Oteo et al., 2018, Raza et al., 2017, Križman et al., 2017, Hassan and Shobrak, 2015, Himsworth et al., 2015, Dias et al., 2014, Martiny et al., 2011) |
| | | | | | | | | |
| SHV-2 | 4 | *E. coli* (ST212, ST4038), *K. pneumoniae* (ST39) | Europe, North America, South America | Argentina, Canada, Czech Republic, Spain | *Chroicocephalus ridibundus, Corvus brachyrhynchos, Corvus corax, Larus dominicanus, Larus michahellis* | - | - | (Jamborova et al., 2018a, Vergara et al., 2017, Liakopoulos et al., 2016, Dolejska et al., 2009) |
| SHV-2a | 2 | *E. coli* (ST617) | South America, North America | Argentina, Canada | *Corvus brachyrhynchos, Larus dominicanus* | - | - | (Liakopoulos et al., 2016, Parker et al., 2016) |
| SHV-5 | 2 | *Citrobacter freundii, E. coli* | Africa, Europe | Algeria, Portugal | *Corvus corax, Tyto alba* | - | Not specified | (Brahmi et al., 2018, Pinto et al., 2010) |
| SHV-7 | 1 | *Enterobacter cloacae* | North America | USA | *Larus delawarensis* | - | - | (Poirel et al., 2012) |
| SHV-12 | 25 | *Aeromonas caviae, Aeromonas hydrophila, Citrobacter freundii, E. coli* (ST10, ST1079, ST1086, ST1128, ST1158, ST117, ST1431, ST155, ST156, ST410, ST453, ST457, ST540, ST542, ST57, ST665, ST746), *K. pneumoniae, Proteus mirabilis, Providencia alcalifaciens* | Europe, North America, Oceania | Australia, Belgium, Croatia, Czech Republic, Germany, Netherlands, Poland, Portugal, Spain, Sweden, Switzerland, USA | *Aquila chrysaetos, Ardea cinereal, Branta canadensis, Branta leucopsis, Buteo buteo, Chroicocephalus novaehollandiae, Chroicocephalus ridibundus, Ciconia Ciconia, Columba livia, Croicocephalus ridibundus, Cuculus canorus, Gyps fulvus, Larus argentatus, Larus canus Larus marinus, Larus michahellis, Milvus milvus, Strix aluco, Sturnus unicolor, Sylvia atricapilla, Tyto alba* | *Capreolus capreolus, Erinaceus europaeus, Lynx pardinus, Martes foina, Meles meles, Rattus norvegicus, Vulpes vulpes* | *Coregonus lavaretus, Mytilu galloprovincialis, Sparus aurata* | (Darwich et al., 2019, Mughini-Gras et al., 2019, Ahlstrom et al., 2019, Alonso et al., 2017a, Dolejska et al., 2018, Atterby et al., 2017, Alonso et al., 2017b, Cristóvão et al., 2017, Vergara et al., 2017, Alcalá et al., 2016, Bonnedahl et al., 2014, Abgottspon et al., 2014, Veldman et al., 2013, Maravić et al., 2013, Guenther et al., 2013b, Zurfluh et al., 2013, Radhouani et al., 2013, Gonçalves et al., 2012, Garmyn et al., 2011, Silva et al., 2011, Wallensten et al., 2011, Literak et al., 2010a, Dolejska et al., 2009, Costa et al., 2006) |

| | | | | | | | | |
|---|---|---|---|---|---|---|---|---|
| SHV-14 | 1 | *K. pneumoniae* | North America | USA | Not specified | - | - | (Bonnedahl et al., 2014) |
| SHV-28 | 2 | *K. pneumoniae* (ST307), *Proteus mirabilis* | Africa, Europe | Guinea, Spain | *Accipiter gentilis, Serinus serinus, Strix aluco* | *Erinaceus europaeus, Rattus rattus* | - | (Darwich et al., 2019, Schaufler et al., 2018) |
| SHV-62 | 2 | *K. pneumoniae* (ST502, ST1211) | Africa | Central African Republic, Guinea | - | *Gorilla gorilla gorilla, Rattus rattus* | - | (Schaufler et al., 2018, Janatova et al., 2014) |
| SHV-102 | 1 | *K. pneumoniae* | North America | USA | Not specified | - | - | (Bonnedahl et al., 2014) |
| SHV-110 | 1 | *K. pneumoniae* (ST377) | Europe | Spain | *Larus michahellis* | - | - | (Ahlstrom et al., 2019) |
| SHV-167 | 1 | *E. coli* | Europe | Spain | *Bubo bubo* | - | - | (Darwich et al., 2019) |
| SHV | 1 | *E. coli* | Europe | Spain | *Anas platyrhynchos, Bubo bubo, Bubulcus ibis, Ciconia ciconia, Coragyps atratus, Larus fuscus, Milvus migrans* | - | - | (Oteo et al., 2018) |
| | | | | | | | | |
| TEM-15 | 1 | *E. coli* (ST23) | Europe | Austria | *Corvus frugilegus* | - | - | (Loncaric et al., 2014) |
| TEM-19 | 1 | *E. coli* (ST2967), *K. pneumoniae* | North America | USA | Not specified | - | - | (Bonnedahl et al., 2014) |
| TEM-20 | 1 | *E. coli* | Europe | Portugal | *Buteo buteo* | - | - | (Pinto et al., 2010) |
| TEM-24 | 1 | *E. coli, Enterobacter cloacae* | Africa | Algeria | - | - | *Pagellus acarne, Sardina pilchardus, Trachurus trachurus* | (Brahmi et al., 2018) |
| TEM-33 | 1 | *E. coli* (ST361) | Asia | Pakistan | *Fulica atra* | - | - | (Mohsin et al., 2017) |
| TEM-52 | 9 | *E. coli* (ST167, ST1844) | Europe, North America | Belgium, Canada, Netherlands, Portugal | *Anser anser domesticus, Larus cachinnans, Larus fuscus, Leucophaeus pipixcan* | Not specified | *Sparus aurata* | (Mughini-Gras et al., 2019, Cristóvão et al., 2017, Monteiro et al., 2016, Bonnedahl et al., 2014, Garmyn et al., 2011, Simões et al., 2010, Poeta et al., 2008, Costa et al., 2008, Costa et al., 2006) |
| TEM-52b | 2 | *E. coli, Enterobacter cloacae* | Africa, Europe | Czech Republic, Senegal | - | *Sus scrofa, Rattus rattus* | - | (Literak et al., 2010b, Ivan Literak et al., 2009) |
| TEM-52c | 1 | *E. coli* | Europe | Netherlands | *Larus argentatus, Philomachus pugnax* | - | - | (Veldman et al., 2013) |
| TEM-84 | 1 | *E. coli* | Europe | Spain | *Larus michahellis* | - | - | (Vergara et al., 2017) |
| TEM-135 | 1 | *E. coli* | Europe | Belgium | *Buteo buteo* | - | - | (Rouffaer et al., 2014) |
| TEM-161 | 1 | *E. coli* | Europe | Poland | - | *Sus scrofa* | - | (Wasyl et al., 2018) |
| TEM-176 | 1 | *E. coli* (ST58, ST361), *Enterobacter xiangfangensis (ST114)* | Europe | Austria | - | *Rattus norvegicus* | - | (Desvars-Larrive et al., 2019) |
| | | | | | | | | |
| PER-1 | 1 | *Aeromonas caviae* | Europe | Croatia | - | - | *Mytilu galloprovincialis* | (Maravić et al., 2013) |

| | | | | | | | | |
|---|---|---|---|---|---|---|---|---|
| PER-2 | 1 | Only ARG | South America | Chile | Not specified | - | - | (Bueno et al., 2020) |
| | | | | | | | | |
| CMY-2 | 31 | *Citrobacter freundii, E. coli* (ST10, ST101, ST131, ST1485, ST162, ST1625, ST167, ST1850, ST195, ST204, ST2064, ST224, ST2721, ST351, ST357, ST3727, ST38, ST410, ST4564, ST540, ST58, ST617, ST68, ST7207, ST7348, ST83, ST93, ST963), *Enterobacter cloacae, K. pneumoniae, Proteus mirabilis, Salmonella enterica* serovar Heidelberg, Only ARG | Africa, Asia, Europe, North America, South America | Argentina, Austria, Brazil, Canada, Denmark, England, France, Italy, Netherlands, Poland, Portugal, Saudi Arabia, Slovakia, Spain, Switzerland, Tunisia, USA | *Accipiter gentilis, Anas platyrhynchos, Caracara plancus, Carduelis carduelis, Chroicocephalus genei, Chroicocephalus ridibundus, Ciconia Ciconia, Corvus brachyrhynchos, Corvus corax, Corvus frugilegus, Cygnus olor, Larus argentatus, Larus delawarensis, Larus dominicanus, Larus fuscus, Larus glaucescens, Larus marinus, Larus michahellis, Rupornis magnirostris, Streptopelia decaocto, Strix aluco, Sylvia melanocephala, Tringa tetanus, Turdus merula, Tyto alba* | *Atelerix algirus, Canis lupus, Capra hircus, Capreolus capreolus, Cervus elaphus, Erinaceus europaeus, Loxodonta Africana, Martes foina, Meles meles, Mustela vison, Oryctolagus cuniculus, Ovis orientalis musimon, Rattus norvegicus, Sus scrofa, Vulpes vulpes* | *Perca fluviatilis* | (Furlan et al., 2020, Flavia Zendri et al., 2020, Bertelloni et al., 2020, Migura-Garcia et al., 2019, Ben Said et al., 2019, Desvars-Larrive et al., 2019, Darwich et al., 2019, Mughini-Gras et al., 2019, Turchi et al., 2019, Sen et al., 2019, Jamborova et al., 2018a, Jamborova et al., 2018b, Batalha de Jesus et al., 2019, Alonso et al., 2017a, Wasyl et al., 2018, Aberkane et al., 2017, Vergara et al., 2017, Liakopoulos et al., 2016, Alcalá et al., 2016, Aberkane et al., 2016, Atterby et al., 2016, Hassan and Shobrak, 2015, Alves et al., 2014, Abgottspon et al., 2014, Loncaric et al., 2014, Veldman et al., 2013, Kmet et al., 2013, Poirel et al., 2012, Martiny et al., 2011, Aarestrup et al., 2004) |
| CMY-16 | 2 | *Salmonella enterica* serovar Corvallis (ST1541) | Europe | Germany | *Milvus migrans* | - | - | (Villa et al., 2015, Fischer et al., 2013) |
| CMY-42 | 1 | *E. coli* | North America | Canada | *Corvus brachyrhynchos, Corvus corax* | - | - | (Jamborova et al., 2018a) |
| CMY-67 | 1 | *Citrobacter freundii* | Europe | Germany | *Passer domesticus* | - | - | (Osieka et al., 2018) |
| CMY-135 | 1 | *Citrobacter freundii* (ST265) | South America | Brazil | - | - | *Chelonia mydas* | (Goldberg et al., 2019) |
| CMY | 7 | *E. coli* (ST1167, ST224, ST405, ST4307, ST744), *Enterobacter asburiae, Proteus mirabilis,* Only ARG | Africa, Europe, North America, South America | Chile, Côte d'Ivoire, Germany, Spain, USA | *Accipiter gentilis, Aix sponsa, Anas acuta, Anas arolinensis, Anas discors, Anas platyrhynchos, Anas strepera, Aquila fasciata, Ardea cinereal, Chroicocephalus ridibundus, Ciconia Ciconia, Coragyps atratus, Cygnus olor, Falco tinnunculus, Gyps fulvus, Larus fuscus, Pernis apivorus* | *Didelphis virginiana, Pan troglodytes verus, Procyon lotor* | - | (Worsley-Tonks et al., 2020, Bueno et al., 2020, Oteo et al., 2018, Mathys et al., 2017b, Mathys et al., 2017a, Schaufler et al., 2016, Albrechtova et al., 2014) |
| | | | | | | | | |
| DHA-1 | 5 | *Citrobacter freundii, K. pneumoniae* (ST11, ST1735, ST502), *Salmonella enterica* serovar Agona (ST13) | Africa, Europe, Oceania | Algeria, Australia, Germany, Guinea, Spain | *Chroicocephalus novaehollandiae, Larus audouinii* | *Ovis orientalis musimon, Rattus rattus* | Not specified | (Brahmi et al., 2018, Schaufler et al., 2018, Papagiannitsis et al., 2017, Loncaric et al., 2016, Antilles et al., 2015) |
| DHA | 2 | *E. coli, Enterobacter asburiae* | Africa, Asia | Côte d'Ivoire, Saudi Arabia | *Gracula religosa* | *Pan troglodytes verus* | - | (Hassan and Shobrak, 2015, Albrechtova et al., 2014) |

| | | | | | | | | |
|---|---|---|---|---|---|---|---|---|
| [redacted] | | | | | | | | |
| ACT-14 | 1 | *E. coli* | Africa | Sudan | - | *Vulpes zerda* | - | (Feng et al., 2019) |
| ACT-16 | 2 | *Enterobacter cloacae* (ST279), *Enterobacter hormaechei* (ST114) | South America | Brazil | - | - | *Bothrops alternatus, Chelonia mydas* | (Goldberg et al., 2019, de Carvalho et al., 2018) |
| ACT-23 | 1 | *Enterobacter cloacae* | Europe | Norway | *Larus hyperboreus* | - | - | (Literak et al., 2014) |
| ACT-36 | 1 | *E. coli, Escherichia fergusonii, Klebsiella oxytoca* | Africa | Tunisia | *Bubulcus ibis, Himantopus himantopus, Nycticorax nycticorax* | - | - | (Ben Yahia et al., 2020) |
| ACT | 3 | *E. coli* (ST224), *Enterobacter asburiae*, Only ARG | Africa, Europe, North America | Côte d'Ivoire, Germany, USA | *Accipiter gentilis* | *Canis latrans, Didelphis virginiana, Pan troglodytes verus, Procyon lotor* | - | (Worsley-Tonks et al., 2020, Schaufler et al., 2016, Albrechtova et al., 2014) |
| [redacted] | | | | | | | | |
| ACC-2 | 1 | *E. coli, Escherichia fergusonii* | Africa | Tunisia | *Bubulcus ibis* | - | - | (Ben Yahia et al., 2020) |
| ACC | 2 | *Hafnia alvei*, Only ARG | Europe, North America | Spain, USA | *Anas platyrhynchos, Apus apus, Aquila fasciata, Buteo buteo, Falco peregrinus* | *Didelphis virginiana* | - | (Worsley-Tonks et al., 2020, Oteo et al., 2018) |
| [redacted] | | | | | | | | |
| FOX-3 | 1 | *Aeromonas caviae* | Europe | Croatia | - | - | *Mytilu galloprovincialis* | (Maravić et al., 2013) |
| FOX-5 | 1 | *K. pneumoniae* | North America | USA | *Larus delawarensis* | - | - | (Poirel et al., 2012) |
| FOX | 1 | *Aeromonas eucrenophila, Aeromonas salmonicida, Aeromonas veronii* | Europe | Portugal | *Circaetus gallicus* | *Cervus elaphus* | *Colubridae* | (Dias et al., 2014) |
| [redacted] | | | | | | | | |
| MOX | 1 | *Aeromonas salmonicida, Aeromonas veronii* | Europe | Portugal | *Circaetus gallicus, Strix aluco* | - | - | (Dias et al., 2014) |
| [redacted] | | | | | | | | |
| BIL | 1 | Only ARG | North America | USA | - | *Didelphis virginiana, Procyon lotor* | - | (Worsley-Tonks et al., 2020) |
| [redacted] | | | | | | | | |
| EBC | 1 | *Citrobacter freundii, Enterobacter asburiae* | Africa | Côte d'Ivoire | - | *Pan troglodytes verus* | - | (Albrechtova et al., 2014) |

## References


AARESTRUP, F. M., HASMAN, H., OLSEN, I. & SORENSEN, G. 2004. International spread of bla(CMY-2)-mediated cephalosporin resistance in a multiresistant Salmonella enterica serovar Heidelberg isolate stemming from the importation of a boar by Denmark from Canada. *Antimicrob Agents Chemother,* 48**,** 1916-7.

ABERKANE, S., COMPAIN, F., DECRÉ, D., DUPONT, C., LAURENS, C., VITTECOQ, M., PANTEL, A., SOLASSOL, J., CARRIÈRE, C., RENAUD, F., BRIEU, N., LAVIGNE, J. P., BOUZINBI, N., OUÉDRAOGO, A. S., JEAN-PIERRE, H. & GODREUIL, S. 2016. High Prevalence of SXT/R391-Related Integrative and Conjugative Elements Carrying blaCMY-2 in Proteus mirabilis Isolates from Gulls in the South of France. *Antimicrob Agents Chemother,* 60**,** 1148-52.

ABERKANE, S., COMPAIN, F., DECRÉ, D., PANTEL, A., VITTECOQ, M., SOLASSOL, J., BOUZINBI, N., JEAN-PIERRE, H. & GODREUIL, S. 2017. Persistence of bla(CMY-2)-producing Proteus mirabilis in two gull colonies at a 1-year interval in Southern France. *J Glob Antimicrob Resist,* 9**,** 138-140.

ABGOTTSPON, H., NÜESCH-INDERBINEN, M. T., ZURFLUH, K., ALTHAUS, D., HÄCHLER, H. & STEPHAN, R. 2014. Enterobacteriaceae with extended-spectrum- and pAmpC-type β-lactamase-encoding genes isolated from freshwater fish from two lakes in Switzerland. *Antimicrob Agents Chemother,* 58**,** 2482-4.

AHLSTROM, C. A., RAMEY, A. M., WOKSEPP, H. & BONNEDAHL, J. 2019. Early emergence of mcr-1-positive Enterobacteriaceae in gulls from Spain and Portugal. *Environ Microbiol Rep,* 11**,** 669-671.

ALBRECHTOVA, K., PAPOUSEK, I., DE NYS, H., PAULY, M., ANOH, E., MOSSOUN, A., DOLEJSKA, M., MASARIKOVA, M., METZGER, S., COUACY-HYMANN, E., AKOUA-KOFFI, C., WITTIG, R. M., KLIMES, J., CIZEK, A., LEENDERTZ, F. H. & LITERAK, I. 2014. Low rates of antimicrobial-resistant Enterobacteriaceae in wildlife in Taï National Park, Côte d'Ivoire, surrounded by villages with high prevalence of multiresistant ESBL-producing Escherichia coli in people and domestic animals. *PLoS One,* 9**,** e113548.

ALCALÁ, L., ALONSO, C. A., SIMÓN, C., GONZÁLEZ-ESTEBAN, C., ORÓS, J., REZUSTA, A., ORTEGA, C. & TORRES, C. 2016. Wild Birds, Frequent Carriers of Extended-Spectrum β-Lactamase (ESBL) Producing Escherichia coli of CTX-M and SHV-12 Types. *Microbial Ecology,* 72**,** 861-869.

ALONSO, C. A., ALCALÁ, L., SIMÓN, C. & TORRES, C. 2017a. Novel sequence types of extended-spectrum and acquired AmpC beta-lactamase producing Escherichia coli and Escherichia clade V isolated from wild mammals. *FEMS Microbiology Ecology,* 93.

ALONSO, C. A., GONZÁLEZ-BARRIO, D., TENORIO, C., RUIZ-FONS, F. & TORRES, C. 2016. Antimicrobial resistance in faecal Escherichia coli isolates from farmed red deer and wild small mammals. Detection of a multiresistant E. coli producing extended-spectrum beta-lactamase. *Comparative Immunology, Microbiology and Infectious Diseases,* 45**,** 34-39.

ALONSO, C. A., MICHAEL, G. B., LI, J., SOMALO, S., SIMÓN, C., WANG, Y., KASPAR, H., KADLEC, K., TORRES, C. & SCHWARZ, S. 2017b. Analysis of blaSHV-12-carrying Escherichia coli clones and plasmids from human, animal and food sources. *Journal of Antimicrobial Chemotherapy,* 72**,** 1589-1596.

ALVES, M. S., PEREIRA, A., ARAÚJO, S. M., CASTRO, B. B., CORREIA, A. C. & HENRIQUES, I. 2014. Seawater is a reservoir of multi-resistant Escherichia coli, including strains hosting plasmid-mediated quinolones resistance and extended-spectrum beta-lactamases genes. *Front Microbiol,* 5**,** 426.

ANTILLES, N., GARCIA-MIGURA, L., JOENSEN, K. G., LEEKITCHAROENPHON, P., AARESTRUP, F. M., CERDÀ-CUÉLLAR, M. & HENDRIKSEN, R. S. 2015. Audouin's gull, a potential vehicle

of an extended spectrum β-lactamase producing Salmonella Agona. *FEMS Microbiol Lett,* 362**,** 1-4.
ATTERBY, C., BÖRJESSON, S., NY, S., JÄRHULT, J. D., BYFORS, S. & BONNEDAHL, J. 2017. ESBL-producing Escherichia coli in Swedish gulls—A case of environmental pollution from humans? *PLOS ONE,* 12**,** e0190380.
ATTERBY, C., RAMEY, A. M., HALL, G. G., JÄRHULT, J., BÖRJESSON, S. & BONNEDAHL, J. 2016. Increased prevalence of antibiotic-resistant E. coli in gulls sampled in Southcentral Alaska is associated with urban environments. *Infection Ecology & Epidemiology,* 6**,** 32334.
BACHIRI, T., BAKOUR, S., LADJOUZI, R., THONGPAN, L., ROLAIN, J. M. & TOUATI, A. 2017. High rates of CTX-M-15-producing Escherichia coli and Klebsiella pneumoniae in wild boars and Barbary macaques in Algeria. *Journal of Global Antimicrobial Resistance,* 8**,** 35-40.
BÁEZ, J., HERNÁNDEZ-GARCÍA, M., GUAMPARITO, C., DÍAZ, S., OLAVE, A., GUERRERO, K., CANTÓN, R., BAQUERO, F., GAHONA, J., VALENZUELA, N., DEL CAMPO, R. & SILVA, J. 2015. Molecular characterization and genetic diversity of ESBL-producing Escherichia coli colonizing the migratory Franklin's gulls (Leucophaeus pipixcan) in Antofagasta, North of Chile. *Microb Drug Resist,* 21**,** 111-6.
BATALHA DE JESUS, A. A., FREITAS, A. A. R., DE SOUZA, J. C., MARTINS, N., BOTELHO, L. A. B., GIRÃO, V. B. C., TEIXEIRA, L. M., RILEY, L. W. & MOREIRA, B. M. 2019. High-Level Multidrug-Resistant Escherichia coli Isolates from Wild Birds in a Large Urban Environment. *Microb Drug Resist,* 25**,** 167-172.
BEN SAID, L., JOUINI, A., FLISS, I., TORRES, C. & KLIBI, N. 2019. Antimicrobial resistance genes and virulence gene encoding intimin in Escherichia coli and Enterococcus isolated from wild rabbits (Oryctolagus cuniculus) in Tunisia. *Acta Vet Hung,* 67**,** 477-488.
BEN YAHIA, H., BEN SALLEM, R., TAYH, G., KLIBI, N., BEN AMOR, I., GHARSA, H., BOUDABBOUS, A. & BEN SLAMA, K. 2018. Detection of CTX-M-15 harboring Escherichia coli isolated from wild birds in Tunisia. *BMC Microbiology,* 18**,** 26.
BEN YAHIA, H., CHAIRAT, S., GHARSA, H., ALONSO, C. A., BEN SALLEM, R., PORRES-OSANTE, N., HAMDI, N., TORRES, C. & BEN SLAMA, K. 2020. First Report of KPC-2 and KPC-3-Producing Enterobacteriaceae in Wild Birds in Africa. *Microbial Ecology,* 79**,** 30-37.
BENAVIDES, J. A., SHIVA, C., VIRHUEZ, M., TELLO, C., APPELGREN, A., VENDRELL, J., SOLASSOL, J., GODREUIL, S. & STREICKER, D. G. 2018. Extended-spectrum beta-lactamase-producing Escherichia coli in common vampire bats Desmodus rotundus and livestock in Peru. *Zoonoses Public Health,* 65**,** 454-458.
BERTELLONI, F., CILIA, G., BOGI, S., EBANI, V. V., TURINI, L., NUVOLONI, R., CERRI, D., FRATINI, F. & TURCHI, B. 2020. Pathotypes and Antimicrobial Susceptibility of Escherichia Coli Isolated from Wild Boar (Sus scrofa) in Tuscany. *Animals : an open access journal from MDPI,* 10**,** 744.
BONNEDAHL, J., DROBNI, M., GAUTHIER-CLERC, M., HERNANDEZ, J., GRANHOLM, S., KAYSER, Y., MELHUS, A., KAHLMETER, G., WALDENSTROM, J., JOHANSSON, A. & OLSEN, B. 2009. Dissemination of Escherichia coli with CTX-M type ESBL between humans and yellow-legged gulls in the south of France. *PLoS One,* 4**,** e5958.
BONNEDAHL, J., DROBNI, P., JOHANSSON, A., HERNANDEZ, J., MELHUS, Å., STEDT, J., OLSEN, B. & DROBNI, M. 2010. Characterization, and comparison, of human clinical and black-headed gull (Larus ridibundus) extended-spectrum β-lactamase-producing bacterial isolates from Kalmar, on the southeast coast of Sweden. *Journal of Antimicrobial Chemotherapy,* 65**,** 1939-1944.
BONNEDAHL, J., HERNANDEZ, J., STEDT, J., WALDENSTRÖM, J., OLSEN, B. & DROBNI, M. 2014. Extended-spectrum β-lactamases in Escherichia coli and Klebsiella pneumoniae in Gulls, Alaska, USA. *Emerg Infect Dis,* 20**,** 897-9.

BONNEDAHL, J., STEDT, J., WALDENSTRÖM, J., SVENSSON, L., DROBNI, M. & OLSEN, B. 2015. Comparison of Extended-Spectrum β-Lactamase (ESBL) CTX-M Genotypes in Franklin Gulls from Canada and Chile. *PLOS ONE,* 10**,** e0141315.
BORGES, C. A., CARDOZO, M. V., BERALDO, L. G., OLIVEIRA, E. S., MALUTA, R. P., BARBOZA, K. B., WERTHER, K. & ÁVILA, F. A. 2017. Wild birds and urban pigeons as reservoirs for diarrheagenic Escherichia coli with zoonotic potential. *Journal of Microbiology,* 55**,** 344-348.
BOUAZIZ, A., LOUCIF, L., AYACHI, A., GUEHAZ, K., BENDJAMA, E. & ROLAIN, J. M. 2018. Migratory White Stork (Ciconia ciconia): A Potential Vector of the OXA-48-Producing Escherichia coli ST38 Clone in Algeria. *Microb Drug Resist,* 24**,** 461-468.
BRAHMI, S., TOUATI, A., DUNYACH-REMY, C., SOTTO, A., PANTEL, A. & LAVIGNE, J. P. 2018. High Prevalence of Extended-Spectrum β-Lactamase-Producing Enterobacteriaceae in Wild Fish from the Mediterranean Sea in Algeria. *Microb Drug Resist,* 24**,** 290-298.
BUENO, I., VERDUGO, C., JIMENEZ-LOPEZ, O., ALVAREZ, P. P., GONZALEZ-ROCHA, G., LIMA, C. A., TRAVIS, D. A., WASS, B., ZHANG, Q., ISHII, S. & SINGER, R. S. 2020. Role of wastewater treatment plants on environmental abundance of Antimicrobial Resistance Genes in Chilean rivers. *International Journal of Hygiene and Environmental Health,* 223**,** 56-64.
COSTA, D., POETA, P., SAENZ, Y., VINUE, L., COELHO, A. C., MATOS, M., ROJO-BEZARES, B., RODRIGUES, J. & TORRES, C. 2008. Mechanisms of antibiotic resistance in Escherichia coli isolates recovered from wild animals. *Microb Drug Resist,* 14**,** 71-7.
COSTA, D., POETA, P., SAENZ, Y., VINUE, L., ROJO-BEZARES, B., JOUINI, A., ZARAZAGA, M., RODRIGUES, J. & TORRES, C. 2006. Detection of Escherichia coli harbouring extended-spectrum beta-lactamases of the CTX-M, TEM and SHV classes in faecal samples of wild animals in Portugal. *J Antimicrob Chemother,* 58**,** 1311-2.
CRISTÓVÃO, F., ALONSO, C. A., IGREJAS, G., SOUSA, M., SILVA, V., PEREIRA, J. E., LOZANO, C., CORTÉS-CORTÉS, G., TORRES, C. & POETA, P. 2017. Clonal diversity of extended-spectrum beta-lactamase producing Escherichia coli isolates in fecal samples of wild animals. *FEMS Microbiology Letters,* 364.
DARWICH, L., VIDAL, A., SEMINATI, C., ALBAMONTE, A., CASADO, A., LÓPEZ, F., MOLINA-LÓPEZ, R. A. & MIGURA-GARCIA, L. 2019. High prevalence and diversity of extended-spectrum β-lactamase and emergence of OXA-48 producing Enterobacterales in wildlife in Catalonia. *PLOS ONE,* 14**,** e0210686.
DE CARVALHO, M. P. N., FERNANDES, M. R., SELLERA, F. P., LOPES, R., MONTE, D. F., HIPPÓLITO, A. G., MILANELO, L., RASO, T. F. & LINCOPAN, N. 2020. International clones of extended-spectrum β-lactamase (CTX-M)-producing Escherichia coli in peri-urban wild animals, Brazil. *Transbound Emerg Dis*.
DE CARVALHO, M. P. N., MOURA, Q., FERNANDES, M. R., SELLERA, F. P., PAGOTTO, A. H., STUGINSKI, D. R., CASTRO, R. A., SANT'ANNA, S. S., GREGO, K. F. & LINCOPAN, N. 2018. Genomic features of a multidrug-resistant Enterobacter cloacae ST279 producing CTX-M-15 and AAC(6')-Ib-cr isolated from fatal infectious stomatitis in a crossed pit viper (Bothrops alternatus). *Journal of Global Antimicrobial Resistance,* 15**,** 290-291.
DENG, Y., WU, Y., JIANG, L., TAN, A., ZHANG, R. & LUO, L. 2016. Multi-Drug Resistance Mediated by Class 1 Integrons in Aeromonas Isolated from Farmed Freshwater Animals. *Frontiers in microbiology,* 7**,** 935-935.
DESVARS-LARRIVE, A., RUPPITSCH, W., LEPUSCHITZ, S., SZOSTAK, M. P., SPERGSER, J., FEßLER, A. T., SCHWARZ, S., MONECKE, S., EHRICHT, R., WALZER, C. & LONCARIC, I. 2019. Urban brown rats (Rattus norvegicus) as possible source of multidrug-resistant Enterobacteriaceae and meticillin-resistant Staphylococcus spp., Vienna, Austria, 2016 and 2017. *Eurosurveillance,* 24**,** 1900149.

DIAS, C., SERRA, C. R., SIMÕES, L. C., SIMÕES, M., MARTINEZ-MURCIA, A. & SAAVEDRA, M. J. 2014. Extended-spectrum β-lactamase and carbapenemase-producing Aeromonas species in wild animals from Portugal. *Vet Rec,* 174**,** 532.
DÍAZ-JIMÉNEZ, D., GARCÍA-MENIÑO, I., HERRERA, A., GARCÍA, V., LÓPEZ-BECEIRO, A. M., ALONSO, M. P., BLANCO, J. & MORA, A. 2020. Genomic Characterization of Escherichia coli Isolates Belonging to a New Hybrid aEPEC/ExPEC Pathotype O153:H10-A-ST10 eae-beta1 Occurred in Meat, Poultry, Wildlife and Human Diarrheagenic Samples. *Antibiotics,* 9**,** 192.
DOLEJSKA, M., BIEROSOVA, B., KOHOUTOVA, L., LITERAK, I. & CIZEK, A. 2009. Antibiotic-resistant Salmonella and Escherichia coli isolates with integrons and extended-spectrum beta-lactamases in surface water and sympatric black-headed gulls. *J Appl Microbiol,* 106**,** 1941-50.
DOLEJSKA, M., PAPAGIANNITSIS, C. C., MEDVECKY, M., DAVIDOVA-GERZOVA, L. & VALCEK, A. 2018. Characterization of the Complete Nucleotide Sequences of IMP-4-Encoding Plasmids, Belonging to Diverse Inc Families, Recovered from <span class="named-content genus-species" id="named-content-1">Enterobacteriaceae</span> Isolates of Wildlife Origin. *Antimicrobial Agents and Chemotherapy,* 62**,** e02434-17.
FENG, C., WEN, P., XU, H., CHI, X., LI, S., YU, X., LIN, X., WU, S. & ZHENG, B. 2019. Emergence and Comparative Genomics Analysis of Extended-Spectrum-β-Lactamase-Producing <span class="named-content genus-species" id="named-content-1">Escherichia coli</span> Carrying <em>mcr-1</em> in Fennec Fox Imported from Sudan to China. *mSphere,* 4**,** e00732-19.
FISCHER, J., SCHMOGER, S., JAHN, S., HELMUTH, R. & GUERRA, B. 2013. NDM-1 carbapenemase-producing Salmonella enterica subsp. enterica serovar Corvallis isolated from a wild bird in Germany. *J Antimicrob Chemother,* 68**,** 2954-6.
FLAVIA ZENDRI, IULIANA E. MACIUCA, SIMON MOON, PHILIP H. JONES, ANDY WATTRET, RICHARD JENKINS, ANDY BAXTER & TIMOFTE, D. 2020. Occurrence of ESBL-Producing Escherichia coli ST131, Including the H30-Rx and C1-M27 Subclones, Among Urban Seagulls from the United Kingdom. *Microbial Drug Resistance,* 26**,** 697-708.
FUENTES-CASTILLO, D., ESPOSITO, F., CARDOSO, B., DALAZEN, G., MOURA, Q., FUGA, B., FONTANA, H., CERDEIRA, L., DROPA, M., ROTTMANN, J., GONZÁLEZ-ACUÑA, D., CATÃO-DIAS, J. L. & LINCOPAN, N. 2020. Genomic data reveal international lineages of critical priority Escherichia coli harbouring wide resistome in Andean condors (Vultur gryphus Linnaeus, 1758). *Molecular ecology,* 29**,** 1919-1935.
FUENTES-CASTILLO, D., FARFÁN-LÓPEZ, M., ESPOSITO, F., MOURA, Q., FERNANDES, M. R., LOPES, R., CARDOSO, B., MUÑOZ, M. E., CERDEIRA, L., NAJLE, I., MUÑOZ, P. M., CATÃO-DIAS, J. L., GONZÁLEZ-ACUÑA, D. & LINCOPAN, N. 2019. Wild owls colonized by international clones of extended-spectrum β-lactamase (CTX-M)-producing Escherichia coli and Salmonella Infantis in the Southern Cone of America. *Sci Total Environ,* 674**,** 554-562.
FUENTES-CASTILLO, D., NAVAS-SUÁREZ, P. E., GONDIM, M. F., ESPOSITO, F., SACRISTÁN, C., FONTANA, H., FUGA, B., PIOVANI, C., KOOIJ, R., LINCOPAN, N. & CATÃO-DIAS, J. L. Genomic characterization of multidrug-resistant ESBL-producing Escherichia coli ST58 causing fatal colibacillosis in critically endangered Brazilian merganser (Mergus octosetaceus). *Transboundary and Emerging Diseases,* n/a.
FURLAN, J. P. R., LOPES, R., GONZALEZ, I. H. L., RAMOS, P. L. & STEHLING, E. G. 2020. Comparative analysis of multidrug resistance plasmids and genetic background of CTX-M-producing Escherichia coli recovered from captive wild animals. *Appl Microbiol Biotechnol,* 104**,** 6707-6717.
GARMYN, A., HAESEBROUCK, F., HELLEBUYCK, T., SMET, A., PASMANS, F., BUTAYE, P. & MARTEL, A. 2011. Presence of extended-spectrum β-lactamase-producing Escherichia coli in wild geese. *Journal of Antimicrobial Chemotherapy,* 66**,** 1643-1644.

GOLDBERG, D. W., FERNANDES, M. R., SELLERA, F. P., COSTA, D. G. C., LOUREIRO BRACARENSE, A. P. & LINCOPAN, N. 2019. Genetic background of CTX-M-15-producing Enterobacter hormaechei ST114 and Citrobacter freundii ST265 co-infecting a free-living green turtle (Chelonia mydas). *Zoonoses and Public Health,* 66**,** 540-545.

GONÇALVES, A., IGREJAS, G., RADHOUANI, H., ESTEPA, V., ALCAIDE, E., ZORRILLA, I., SERRA, R., TORRES, C. & POETA, P. 2012. Detection of extended-spectrum beta-lactamase-producing Escherichia coli isolates in faecal samples of Iberian lynx. *Letters in Applied Microbiology,* 54**,** 73-77.

GONÇALVES, A., POETA, P., MONTEIRO, R., MARINHO, C., SILVA, N., GUERRA, A., PETRUCCI-FONSECA, F., RODRIGUES, J., TORRES, C., VITORINO, R., DOMINGUES, P. & IGREJAS, G. 2014. Comparative proteomics of an extended spectrum β-lactamase producing Escherichia coli strain from the Iberian wolf. *J Proteomics,* 104**,** 80-93.

GUENTHER, S., ASCHENBRENNER, K., STAMM, I., BETHE, A., SEMMLER, T., STUBBE, A., STUBBE, M., BATSAJKHAN, N., GLUPCZYNSKI, Y., WIELER, L. H. & EWERS, C. 2013a. Comparable High Rates of Extended-Spectrum-Beta-Lactamase-Producing Escherichia coli in Birds of Prey from Germany and Mongolia. *PLOS ONE,* 7**,** e53039.

GUENTHER, S., GROBBEL, M., BEUTLICH, J., BETHE, A., FRIEDRICH, N. D., GOEDECKE, A., LÜBKE-BECKER, A., GUERRA, B., WIELER, L. H. & EWERS, C. 2010a. CTX-M-15-type extended-spectrum beta-lactamases-producing Escherichia coli from wild birds in Germany. *Environmental Microbiology Reports,* 2**,** 641-645.

GUENTHER, S., GROBBEL, M., BEUTLICH, J., GUERRA, B., ULRICH, R. G., WIELER, L. H. & EWERS, C. 2010b. Detection of pandemic B2-O25-ST131 Escherichia coli harbouring the CTX-M-9 extended-spectrum beta-lactamase type in a feral urban brown rat (Rattus norvegicus). *J Antimicrob Chemother,* 65**,** 582-4.

GUENTHER, S., SEMMLER, T., STUBBE, A., STUBBE, M., WIELER, L. H. & SCHAUFLER, K. 2017. Chromosomally encoded ESBL genes in Escherichia coli of ST38 from Mongolian wild birds. *Journal of Antimicrobial Chemotherapy,* 72**,** 1310-1313.

GUENTHER, S., WUTTKE, J., BETHE, A., VOJTĚCH, J., SCHAUFLER, K., SEMMLER, T., ULRICH, R. G., WIELER, L. H. & EWERS, C. 2013b. Is Fecal Carriage of Extended-Spectrum-β-Lactamase-Producing <span class="named-content genus-species" id="named-content-1">Escherichia coli</span> in Urban Rats a Risk for Public Health? *Antimicrobial Agents and Chemotherapy,* 57**,** 2424-2425.

HASAN, B., LAURELL, K., RAKIB, M. M., AHLSTEDT, E., HERNANDEZ, J., CACERES, M. & JÄRHULT, J. D. 2016. Fecal Carriage of Extended-Spectrum β-Lactamases in Healthy Humans, Poultry, and Wild Birds in León, Nicaragua-A Shared Pool of bla(CTX-M) Genes and Possible Interspecies Clonal Spread of Extended-Spectrum β-Lactamases-Producing Escherichia coli. *Microb Drug Resist,* 22**,** 682-687.

HASAN, B., MELHUS, Å., SANDEGREN, L., ALAM, M. & OLSEN, B. 2014. The gull (Chroicocephalus brunnicephalus) as an environmental bioindicator and reservoir for antibiotic resistance on the coastlines of the Bay of Bengal. *Microb Drug Resist,* 20**,** 466-71.

HASAN, B., OLSEN, B., ALAM, A., AKTER, L. & MELHUS, Å. 2015. Dissemination of the multidrug-resistant extended-spectrum β-lactamase-producing Escherichia coli O25b-ST131 clone and the role of house crow (Corvus splendens) foraging on hospital waste in Bangladesh. *Clinical Microbiology and Infection,* 21**,** 1000.e1-1000.e4.

HASAN, B., SANDEGREN, L., MELHUS, A., DROBNI, M., HERNANDEZ, J., WALDENSTRÖM, J., ALAM, M. & OLSEN, B. 2012. Antimicrobial drug-resistant Escherichia coli in wild birds and free-range poultry, Bangladesh. *Emerg Infect Dis,* 18**,** 2055-8.

HASSAN, S. A. & SHOBRAK, M. Y. 2015. Detection of genes mediating beta-lactamase production in isolates of enterobacteria recovered from wild pets in Saudi Arabia. *Vet World,* 8**,** 1400-4.

HATHCOCK, T., POUDEL, A., KANG, Y., BUTAYE, P., RAIFORD, D., MOBLEY, T., WANG, C. & BELLAH, J. 2019. Multidrug-Resistant Escherichia coli and Tetracycline-Resistant Enterococcus faecalis in Wild Raptors of Alabama and Georgia, USA. *J Wildl Dis,* 55**,** 482-487.

HERNANDEZ, J., BONNEDAHL, J., ELIASSON, I., WALLENSTEN, A., COMSTEDT, P., JOHANSSON, A., GRANHOLM, S., MELHUS, Å., OLSEN, B. & DROBNI, M. 2010. Globally disseminated human pathogenic Escherichia coli of O25b-ST131 clone, harbouring blaCTX-M-15, found in Glaucous-winged gull at remote Commander Islands, Russia. *Environmental Microbiology Reports,* 2**,** 329-332.

HERNANDEZ, J., JOHANSSON, A., STEDT, J., BENGTSSON, S., PORCZAK, A., GRANHOLM, S., GONZÁLEZ-ACUÑA, D., OLSEN, B., BONNEDAHL, J. & DROBNI, M. 2013. Characterization and Comparison of Extended-Spectrum β-Lactamase (ESBL) Resistance Genotypes and Population Structure of Escherichia coli Isolated from Franklin's Gulls (Leucophaeus pipixcan) and Humans in Chile. *PLOS ONE,* 8**,** e76150.

HESSMAN, J., ATTERBY, C., OLSEN, B. & JÄRHULT, J. D. 2018. High Prevalence and Temporal Variation of Extended Spectrum β-Lactamase-Producing Bacteria in Urban Swedish Mallards. *Microb Drug Resist,* 24**,** 822-829.

HIMSWORTH, C. G., ZABEK, E., DESRUISSEAU, A., PARMLEY, E. J., REID-SMITH, R., JARDINE, C. M., TANG, P. & PATRICK, D. M. 2015. PREVALENCE AND CHARACTERISTICS OF ESCHERICHIA COLI AND SALMONELLA SPP. IN THE FECES OF WILD URBAN NORWAY AND BLACK RATS (RATTUS NORVEGICUS AND RATTUS RATTUS) FROM AN INNER-CITY NEIGHBORHOOD OF VANCOUVER, CANADA. *J Wildl Dis,* 51**,** 589-600.

HO, P. L., CHAN, J., LO, W. U., LAW, P. Y., LI, Z., LAI, E. L. & CHOW, K. H. 2013. Dissemination of plasmid-mediated fosfomycin resistance fosA3 among multidrug-resistant Escherichia coli from livestock and other animals. *Journal of Applied Microbiology,* 114**,** 695-702.

IVAN LITERAK, MONIKA DOLEJSKA, ALOIS CIZEK, CHIEKH AHMED TIDIANE DJIGO, KONECNY, A. & KOUBEK, P. 2009. Reservoirs of antibiotic-resistant Enterobacteriaceae among animals sympatric to humans in Senegal: Extended-spectrum beta-lactamases in bacteria in a black rat (Rattus rattus). *African Journal of Microbiology Research,* 3**,** 751-754.

JAMBOROVA, I., JANECKO, N., HALOVA, D., SEDMIK, J., MEZEROVA, K., PAPOUSEK, I., KUTILOVA, I., DOLEJSKA, M., CIZEK, A. & LITERAK, I. 2018a. Molecular characterization of plasmid-mediated AmpC beta-lactamase- and extended-spectrum beta-lactamase-producing Escherichia coli and Klebsiella pneumoniae among corvids (Corvus brachyrhynchos and Corvus corax) roosting in Canada. *FEMS Microbiology Ecology,* 94.

JAMBOROVA, I., JOHNSTON, B. D., PAPOUSEK, I., KACHLIKOVA, K., MICENKOVA, L., CLABOTS, C., SKALOVA, A., CHUDEJOVA, K., DOLEJSKA, M., LITERAK, I. & JOHNSON, J. R. 2018b. Extensive Genetic Commonality among Wildlife, Wastewater, Community, and Nosocomial Isolates of <span class="named-content genus-species" id="named-content-1">Escherichia coli</span> Sequence Type 131 (<em>H</em>30R1 and <em>H</em>30Rx Subclones) That Carry <em>bla</em><sub>CTX-M-27</sub> or <em>bla</em><sub>CTX-M-15</sub>. *Antimicrobial Agents and Chemotherapy,* 62**,** e00519-18.

JANATOVA, M., ALBRECHTOVA, K., PETRZELKOVA, K. J., DOLEJSKA, M., PAPOUSEK, I., MASARIKOVA, M., CIZEK, A., TODD, A., SHUTT, K., KALOUSOVA, B., PROFOUSOVA-PSENKOVA, I., MODRY, D. & LITERAK, I. 2014. Antimicrobial-resistant Enterobacteriaceae from humans and wildlife in Dzanga-Sangha Protected Area, Central African Republic. *Vet Microbiol,* 171**,** 422-31.

JONES-DIAS, D., MANAGEIRO, V., GRAÇA, R., SAMPAIO, D. A., ALBUQUERQUE, T., THEMUDO, P., VIEIRA, L., FERREIRA, E., CLEMENTE, L. & CANIÇA, M. 2016. QnrS1- and Aac(6′)-Ib-cr-Producing Escherichia coli among Isolates from Animals of Different Sources: Susceptibility and Genomic Characterization. *Frontiers in Microbiology,* 7.

KLIMES, J., MACHALKOVA, M., DOLEJSKA, M., CIZEK, A., JANOSZOWSKA, D., ALEXA, P., ALBRECHTOVA, K., VOJTECH, J. & LITERAK, I. 2013. Escherichia coli-producing extended-spectrum beta-lactamase CTX-M-15 in a captive South American tapir (Tapirus terrestris). *J Zoo Wildl Med,* 44**,** 173-5.

KMET, V., DRUGDOVA, Z., KMETOVA, M. & STANKO, M. 2013. Virulence and antibiotic resistance of Escherichia coli isolated from rooks. *Annals of Agricultural and Environmental Medicine,* 20**,** 273-275.

KRIŽMAN, M., KIRBIŠ, A. & JAMNIKAR-CIGLENEČKI, U. 2017. Antimicrobial-resistant bacteria in wild game in Slovenia. *IOP Conference Series: Earth and Environmental Science,* 85**,** 012083.

LIAKOPOULOS, A., OLSEN, B., GEURTS, Y., ARTURSSON, K., BERG, C., MEVIUS, D. J. & BONNEDAHL, J. 2016. Molecular Characterization of Extended-Spectrum-Cephalosporin-Resistant Enterobacteriaceae from Wild Kelp Gulls in South America. *Antimicrob Agents Chemother,* 60**,** 6924-6927.

LITERAK, I., DOLEJSKA, M., JANOSZOWSKA, D., HRUSAKOVA, J., MEISSNER, W., RZYSKA, H., BZOMA, S. & CIZEK, A. 2010a. Antibiotic-Resistant <em>Escherichia coli</em> Bacteria, Including Strains with Genes Encoding the Extended-Spectrum Beta-Lactamase and QnrS, in Waterbirds on the Baltic Sea Coast of Poland. *Applied and Environmental Microbiology,* 76**,** 8126-8134.

LITERAK, I., DOLEJSKA, M., RADIMERSKY, T., KLIMES, J., FRIEDMAN, M., AARESTRUP, F. M., HASMAN, H. & CIZEK, A. 2010b. Antimicrobial-resistant faecal Escherichia coli in wild mammals in central Europe: multiresistant Escherichia coli producing extended-spectrum beta-lactamases in wild boars. *Journal of Applied Microbiology,* 108**,** 1702-1711.

LITERAK, I., MANGA, I., WOJCZULANIS-JAKUBAS, K., CHROMA, M., JAMBOROVA, I., DOBIASOVA, H., SEDLAKOVA, M. H. & CIZEK, A. 2014. Enterobacter cloacae with a novel variant of ACT AmpC beta-lactamase originating from glaucous gull (Larus hyperboreus) in Svalbard. *Vet Microbiol,* 171**,** 432-5.

LONCARIC, I., BEIGLBÖCK, C., FEßLER, A. T., POSAUTZ, A., ROSENGARTEN, R., WALZER, C., EHRICHT, R., MONECKE, S., SCHWARZ, S., SPERGSER, J. & KÜBBER-HEISS, A. 2016. Characterization of ESBL- and AmpC-Producing and Fluoroquinolone-Resistant Enterobacteriaceae Isolated from Mouflons (Ovis orientalis musimon) in Austria and Germany. *PloS one,* 11**,** e0155786-e0155786.

LONCARIC, I., STALDER, G. L., MEHINAGIC, K., ROSENGARTEN, R., HOELZL, F., KNAUER, F. & WALZER, C. 2014. Comparison of ESBL – And AmpC Producing Enterobacteriaceae and Methicillin-Resistant Staphylococcus aureus (MRSA) Isolated from Migratory and Resident Population of Rooks (Corvus frugilegus) in Austria. *PLOS ONE,* 8**,** e84048.

MAIRI, A., PANTEL, A., OUSALEM, F., SOTTO, A., TOUATI, A. & LAVIGNE, J.-P. 2019. OXA-48-producing Enterobacterales in different ecological niches in Algeria: clonal expansion, plasmid characteristics and virulence traits. *Journal of Antimicrobial Chemotherapy,* 74**,** 1848-1855.

MARAVIĆ, A., SKOČIBUŠIĆ, M., ŠAMANIĆ, I., FREDOTOVIĆ, Ž., CVJETAN, S., JUTRONIĆ, M. & PUIZINA, J. 2013. Aeromonas spp. simultaneously harbouring blaCTX-M-15, blaSHV-12, blaPER-1 and blaFOX-2, in wild-growing Mediterranean mussel (Mytilus galloprovincialis) from Adriatic Sea, Croatia. *International Journal of Food Microbiology,* 166**,** 301-308.

MARCOS PAULO VIEIRA, C., MIRELA CAROLINE VILELA, O., MARIA GABRIELA XAVIER, O., MARCIA CRISTINA, M. & TEREZINHA, K. 2019. CTX-M-producing Escherichia coli Isolated from urban pigeons (Columba livia domestica) in Brazil. *The Journal of Infection in Developing Countries,* 13.

MARTINY, A., MARTINY, J., WEIHE, C., FIELD, A. & ELLIS, J. 2011. Functional Metagenomics Reveals Previously Unrecognized Diversity of Antibiotic Resistance Genes in Gulls. *Frontiers in Microbiology,* 2.

MATEUS-VARGAS, R. H., ATANASSOVA, V., REICH, F. & KLEIN, G. 2017. Antimicrobial susceptibility and genetic characterization of Escherichia coli recovered from frozen game meat. *Food Microbiology,* 63**,** 164-169.

MATHYS, D. A., MATHYS, B. A., MOLLENKOPF, D. F., DANIELS, J. B. & WITTUM, T. E. 2017a. Enterobacteriaceae Harboring AmpC (bla(CMY)) and ESBL (bla(CTX-M)) in Migratory and Nonmigratory Wild Songbird Populations on Ohio Dairies. *Vector Borne Zoonotic Dis,* 17**,** 254-259.

MATHYS, D. A., MOLLENKOPF, D. F., NOLTING, J., BOWMAN, A. S., DANIELS, J. B. & WITTUM, T. E. 2017b. Extended-Spectrum Cephalosporin-Resistant Enterobacteriaceae in Enteric Microflora of Wild Ducks. *J Wildl Dis,* 53**,** 690-694.

MBEHANG NGUEMA, P. P., ONANGA, R., NDONG ATOME, G. R., OBAGUE MBEANG, J. C., MABIKA MABIKA, A., YARO, M., LOUNNAS, M., DUMONT, Y., ZOHRA, Z. F., GODREUIL, S. & BRETAGNOLLE, F. 2020. Characterization of ESBL-Producing Enterobacteria from Fruit Bats in an Unprotected Area of Makokou, Gabon. *Microorganisms,* 8.

MIGURA-GARCIA, L., GONZÁLEZ-LÓPEZ, J. J., MARTINEZ-URTAZA, J., AGUIRRE SÁNCHEZ, J. R., MORENO-MINGORANCE, A., PEREZ DE ROZAS, A., HÖFLE, U., RAMIRO, Y. & GONZALEZ-ESCALONA, N. 2019. mcr-Colistin Resistance Genes Mobilized by IncX4, IncHI2, and IncI2 Plasmids in Escherichia coli of Pigs and White Stork in Spain. *Front Microbiol,* 10**,** 3072.

MOHSIN, M., RAZA, S., SCHAUFLER, K., ROSCHANSKI, N., SARWAR, F., SEMMLER, T., SCHIERACK, P. & GUENTHER, S. 2017. High Prevalence of CTX-M-15-Type ESBL-Producing E. coli from Migratory Avian Species in Pakistan. *Frontiers in Microbiology,* 8.

MONTEIRO, R., HÉBRAUD, M., CHAFSEY, I., POETA, P. & IGREJAS, G. 2016. How different is the proteome of the extended spectrum β-lactamase producing Escherichia coli strains from seagulls of the Berlengas natural reserve of Portugal? *J Proteomics,* 145**,** 167-176.

MUGHINI-GRAS, L., DORADO-GARCÍA, A., VAN DUIJKEREN, E., VAN DEN BUNT, G., DIERIKX, C. M., BONTEN, M. J. M., BOOTSMA, M. C. J., SCHMITT, H., HALD, T., EVERS, E. G., DE KOEIJER, A., VAN PELT, W., FRANZ, E., MEVIUS, D. J. & HEEDERIK, D. J. J. 2019. Attributable sources of community-acquired carriage of Escherichia coli containing β-lactam antibiotic resistance genes: a population-based modelling study. *The Lancet Planetary Health,* 3**,** e357-e369.

NGAIGANAM, E. P., PAGNIER, I., CHAALAL, W., LEANGAPICHART, T., CHABOU, S., ROLAIN, J.-M. & DIENE, S. M. 2019. Investigation of urban birds as source of β-lactamase-producing Gram-negative bacteria in Marseille city, France. *Acta Veterinaria Scandinavica,* 61**,** 51.

NOWAKIEWICZ, A., ZIĘBA, P., GNAT, S., TROŚCIAŃCZYK, A., OSIŃSKA, M., ŁAGOWSKI, D., KOSIOR-KORZECKA, U. & PUZIO, I. 2020. Bats as a reservoir of resistant Escherichia coli: A methodical view. Can we fully estimate the scale of resistance in the reservoirs of free-living animals? *Res Vet Sci,* 128**,** 49-58.

OSIEKA, V., GROBBEL, M., SCHMOGER, S., SZENTIKS, C. A., IRRGANG, A., KÄSBOHRER, A., TENHAGEN, B.-A. & HAMMERL, J. A. 2018. Complete Draft Genome Sequence of an Extended-Spectrum β-Lactamase-Producing <span class="named-content genus-species" id="named-content-1">Citrobacter freundii</span> Strain Recovered from the Intestine of a House Sparrow (<span class="named-content genus-species" id="named-content-2">Passer domesticus</span>) in Germany, 2017. *Genome Announcements,* 6**,** e00599-18.

OTEO, J., MENCÍA, A., BAUTISTA, V., PASTOR, N., LARA, N., GONZÁLEZ-GONZÁLEZ, F., GARCÍA-PEÑA, F. J. & CAMPOS, J. 2018. Colonization with Enterobacteriaceae-Producing ESBLs, AmpCs, and OXA-48 in Wild Avian Species, Spain 2015-2016. *Microb Drug Resist,* 24**,** 932-938.

PAPAGIANNITSIS, C. C., KUTILOVA, I., MEDVECKY, M., HRABAK, J. & DOLEJSKA, M. 2017. Characterization of the Complete Nucleotide Sequences of IncA/C<sub>2</sub> Plasmids Carrying In809-Like Integrons from <span class="named-content genus-species" id="named-content-1">Enterobacteriaceae</span> Isolates of Wildlife Origin. *Antimicrobial Agents and Chemotherapy,* 61**,** e01093-17.

PARKER, D., SNIATYNSKI, M. K., MANDRUSIAK, D. & RUBIN, J. E. 2016. Extended-spectrum β-lactamase producing Escherichia coli isolated from wild birds in Saskatoon, Canada. *Letters in Applied Microbiology,* 63**,** 11-15.

PINTO, L., RADHOUANI, H., COELHO, C., MARTINS DA COSTA, P., SIMÕES, R., BRANDÃO, R. M. L., TORRES, C., IGREJAS, G. & POETA, P. 2010. Genetic Detection of Extended-Spectrum β-Lactamase-Containing <em>Escherichia coli</em> Isolates from Birds of Prey from Serra da Estrela Natural Reserve in Portugal. *Applied and Environmental Microbiology,* 76**,** 4118-4120.

POETA, P., RADHOUANI, H., IGREJAS, G., GONCALVES, A., CARVALHO, C., RODRIGUES, J., VINUE, L., SOMALO, S. & TORRES, C. 2008. Seagulls of the Berlengas natural reserve of Portugal as carriers of fecal Escherichia coli harboring CTX-M and TEM extended-spectrum beta-lactamases. *Appl Environ Microbiol,* 74**,** 7439-41.

POETA, P., RADHOUANI, H., PINTO, L., MARTINHO, A., REGO, V., RODRIGUES, R., GONCALVES, A., RODRIGUES, J., ESTEPA, V., TORRES, C. & IGREJAS, G. 2009. Wild boars as reservoirs of extended-spectrum beta-lactamase (ESBL) producing Escherichia coli of different phylogenetic groups. *J Basic Microbiol,* 49**,** 584-8.

POIREL, L., POTRON, A., DE LA CUESTA, C., CLEARY, T., NORDMANN, P. & MUNOZ-PRICE, L. S. 2012. Wild Coastline Birds as Reservoirs of Broad-Spectrum-β-Lactamase-Producing <span class="named-content genus-species" id="named-content-1">Enterobacteriaceae</span> in Miami Beach, Florida. *Antimicrobial Agents and Chemotherapy,* 56**,** 2756-2758.

RADHOUANI, H., IGREJAS, G., GONÇALVES, A., ESTEPA, V., SARGO, R., TORRES, C. & POETA, P. 2013. Molecular characterization of extended-spectrum-beta-lactamase-producing Escherichia coli isolates from red foxes in Portugal. *Archives of Microbiology,* 195**,** 141-144.

RADHOUANI, H., PINTO, L., COELHO, C., GONCALVES, A., SARGO, R., TORRES, C., IGREJAS, G. & POETA, P. 2010. Detection of Escherichia coli harbouring extended-spectrum {beta}-lactamases of the CTX-M classes in faecal samples of common buzzards (Buteo buteo). *J Antimicrob Chemother,* 65**,** 171-3.

RASHID, M., RAKIB, M. M. & HASAN, B. 2015. Antimicrobial-resistant and ESBL-producing Escherichia coli in different ecological niches in Bangladesh. *Infection Ecology & Epidemiology,* 5**,** 26712.

RAZA, S., MOHSIN, M., MADNI, W. A., SARWAR, F., SAQIB, M. & ASLAM, B. 2017. First Report of blaCTX-M-15-Type ESBL-Producing Klebsiella pneumoniae in Wild Migratory Birds in Pakistan. *EcoHealth,* 14**,** 182-186.

ROUFFAER, L. O., HAESEBROUCK, F. & MARTEL, A. 2014. Extended-spectrum β-lactamase-producing Enterobacteriaceae isolated from feces of Falconidae, Accipitridae, and Laridae in bird rescue centers in Belgium. *J Wildl Dis,* 50**,** 957-60.

SACRISTÁN, I., ESPERÓN, F., ACUÑA, F., AGUILAR, E., GARCÍA, S., LÓPEZ, M. J., CEVIDANES, A., NEVES, E., CABELLO, J., HIDALGO-HERMOSO, E., POULIN, E., MILLÁN, J. & NAPOLITANO, C. 2020. Antibiotic resistance genes as landscape anthropization indicators: Using a wild felid as sentinel in Chile. *Sci Total Environ,* 703**,** 134900.

SCHAUFLER, K., NOWAK, K., DÜX, A., SEMMLER, T., VILLA, L., KOUROUMA, L., BANGOURA, K., WIELER, L. H., LEENDERTZ, F. H. & GUENTHER, S. 2018. Clinically Relevant ESBL-Producing K. pneumoniae ST307 and E. coli ST38 in an Urban West African Rat Population. *Front Microbiol,* 9**,** 150.

SCHAUFLER, K., SEMMLER, T., WIELER, L. H., WÖHRMANN, M., BADDAM, R., AHMED, N., MÜLLER, K., KOLA, A., FRUTH, A., EWERS, C. & GUENTHER, S. 2016. Clonal spread and interspecies transmission of clinically relevant ESBL-producing Escherichia coli of ST410--another successful pandemic clone? *FEMS Microbiol Ecol,* 92.

SELLERA, F. P., FERNANDES, M. R., SARTORI, L., CARVALHO, M. P., ESPOSITO, F., NASCIMENTO, C. L., DUTRA, G. H., MAMIZUKA, E. M., PÉREZ-CHAPARRO, P. J., MCCULLOCH, J. A. & LINCOPAN, N. 2017. Escherichia coli carrying IncX4 plasmid-mediated mcr-1 and blaCTX-M genes in infected migratory Magellanic penguins (Spheniscus magellanicus). *J Antimicrob Chemother,* 72**,** 1255-1256.

SEN, K., BERGLUND, T., SOARES, M. A., TAHERI, B., MA, Y., KHALIL, L., FRIDGE, M., LU, J. & TURNER, R. J. 2019. Antibiotic Resistance of E. coli Isolated From a Constructed Wetland Dominated by a Crow Roost, With Emphasis on ESBL and AmpC Containing E. coli. *Front Microbiol,* 10**,** 1034.

SILVA, N., IGREJAS, G., RODRIGUES, P., RODRIGUES, T., GONÇALVES, A., FELGAR, A. C., PACHECO, R., GONÇALVES, D., CUNHA, R. & POETA, P. 2011. Molecular characterization of vancomycin-resistant enterococci and extended-spectrum β-lactamase-containing Escherichia coli isolates in wild birds from the Azores Archipelago. *Avian Pathology,* 40**,** 473-479.

SIMÕES, R., FERREIRA, C., GONÇALVES, J., ÁLVARES, F., RIO-MAIOR, H., ROQUE, S., BRANDÃO, R. & MARTINS DA COSTA, P. 2012. Occurrence of virulence genes in multidrug-resistant Escherichia coli isolates from Iberian wolves (Canis lupus signatus) in Portugal. *European Journal of Wildlife Research,* 58**,** 677-684.

SIMÕES, R. R., POIREL, L., DA COSTA, P. M. & NORDMANN, P. 2010. Seagulls and beaches as reservoirs for multidrug-resistant Escherichia coli. *Emerging infectious diseases,* 16**,** 110-112.

SÖDERLUND, R., SKARIN, H., BÖRJESSON, S., SANNÖ, A., JERNBERG, T., ASPÁN, A., ÅGREN, E. O. & HANSSON, I. 2019. Prevalence and genomic characteristics of zoonotic gastro-intestinal pathogens and ESBL/pAmpC producing Enterobacteriaceae among Swedish corvid birds. *Infect Ecol Epidemiol,* 9**,** 1701399.

STEDT, J., BONNEDAHL, J., HERNANDEZ, J., WALDENSTRÖM, J., MCMAHON, B. J., TOLF, C., OLSEN, B. & DROBNI, M. 2015. Carriage of CTX-M type extended spectrum β-lactamases (ESBLs) in gulls across Europe. *Acta Vet Scand,* 57**,** 74.

STEPHAN, R. & HÄCHLER, H. 2012. Discovery of extended-spectrum beta-lactamase producing Escherichia coli among hunted deer, chamois and ibex. *Schweiz Arch Tierheilkd,* 154**,** 475-8.

TAOUS BACHIRI, RYM LALAOUI, SOFIANE BAKOUR, MERIEM ALLOUACHE, NADIA BELKEBLA, JEAN MARC ROLAIN & TOUATI, A. 2018. First Report of the Plasmid-Mediated Colistin Resistance Gene mcr-1 in Escherichia coli ST405 Isolated from Wildlife in Bejaia, Algeria. *Microbial Drug Resistance,* 24**,** 890-895.

TIMONIN, M. E., POISSANT, J., MCLOUGHLIN, P. D., HEDLIN, C. E. & RUBIN, J. E. 2017. A survey of the antimicrobial susceptibility of Escherichia coli isolated from Sable Island horses. *Can J Microbiol,* 63**,** 246-251.

TURCHI, B., DEC, M., BERTELLONI, F., WINIARCZYK, S., GNAT, S., BRESCIANI, F., VIVIANI, F., CERRI, D. & FRATINI, F. 2019. Antibiotic Susceptibility and Virulence Factors in Escherichia coli from Sympatric Wildlife of the Apuan Alps Regional Park (Tuscany, Italy). *Microb Drug Resist,* 25**,** 772-780.

VELDMAN, K., VAN TULDEN, P., KANT, A., TESTERINK, J. & MEVIUS, D. 2013. Characteristics of cefotaxime resistant <em>E. coli</em> from wild birds in The Netherlands. *Applied and Environmental Microbiology***,** AEM.01880-13.

VELHNER, M., TODOROVIĆ, D., GREGO, E., JOVČIĆ, B., PRUNIĆ, B., STOJANOV, I. & KEHRENBERG, C. 2018. Fluoroquinolone-resistant and extended-spectrum beta-

lactamase producing Escherichia coli isolates from free-living wild animals. *Vet Microbiol,* 223**,** 168-172.
VERGARA, A., PITART, C., MONTALVO, T., ROCA, I., SABATÉ, S., HURTADO, J. C., PLANELL, R., MARCO, F., RAMÍREZ, B., PERACHO, V., DE SIMÓN, M. & VILA, J. 2017. Prevalence of Extended-Spectrum-β-Lactamase- and/or Carbapenemase-Producing Escherichia coli Isolated from Yellow-Legged Gulls from Barcelona, Spain. *Antimicrob Agents Chemother,* 61.
VILLA, L., GUERRA, B., SCHMOGER, S., FISCHER, J., HELMUTH, R., ZONG, Z., GARCÍA-FERNÁNDEZ, A. & CARATTOLI, A. 2015. IncA/C Plasmid Carrying bla(NDM-1), bla(CMY-16), and fosA3 in a Salmonella enterica Serovar Corvallis Strain Isolated from a Migratory Wild Bird in Germany. *Antimicrob Agents Chemother,* 59**,** 6597-600.
WALLENSTEN, A., HERNANDEZ, J., ARDILES, K., GONZÁLEZ-ACUÑA, D., DROBNI, M. & OLSEN, B. 2011. Extended spectrum beta-lactamases detected in Escherichia coli from gulls in Stockholm, Sweden. *Infection Ecology & Epidemiology,* 1**,** 7030.
WASYL, D., ZAJĄC, M., LALAK, A., SKARŻYŃSKA, M., SAMCIK, I., KWIT, R., JABŁOŃSKI, A., BOCIAN, Ł., WOŹNIAKOWSKI, G., HOSZOWSKI, A. & SZULOWSKI, K. 2018. Antimicrobial Resistance in Escherichia coli Isolated from Wild Animals in Poland. *Microb Drug Resist,* 24**,** 807-815.
WORSLEY-TONKS, K. E. L., MILLER, E. A., GEHRT, S. D., MCKENZIE, S. C., TRAVIS, D. A., JOHNSON, T. J. & CRAFT, M. E. 2020. Characterization of antimicrobial resistance genes in Enterobacteriaceae carried by suburban mesocarnivores and locally owned and stray dogs. *Zoonoses and Public Health,* 67**,** 460-466.
YıLMAZ, E. Ş. & DOLAR, A. 2017. Detection of Extended-Spectrum β-Lactamases in Escherichia coli From Cage Birds. *Journal of Exotic Pet Medicine,* 26**,** 13-18.
ZHOU, W., ZHU, L., JIA, M., WANG, T., LIANG, B., JI, X., SUN, Y., LIU, J. & GUO, X. 2018. Detection of Multi-Drug-Resistant Escherichia coli in a Giant Panda ( Ailuropoda melanoleuca) with Extraintestinal Polyinfection. *J Wildl Dis,* 54**,** 626-630.
ZOU, H., ZHENG, B., SUN, M., OTTOSON, J., LI, Y., BERGLUND, B., CHI, X., JI, X., LI, X., STÅLSBY LUNDBORG, C. & NILSSON, L. E. 2019. Evaluating Dissemination Mechanisms of Antibiotic-Resistant Bacteria in Rural Environments in China by Using CTX-M-Producing Escherichia coli as an Indicator. *Microb Drug Resist,* 25**,** 975-984.
ZURFLUH, K., ALBINI, S., MATTMANN, P., KINDLE, P., NÜESCH-INDERBINEN, M., STEPHAN, R. & VOGLER, B. R. 2019. Antimicrobial resistant and extended-spectrum β-lactamase producing Escherichia coli in common wild bird species in Switzerland. *MicrobiologyOpen,* 8**,** e845.
ZURFLUH, K., NÜESCH-INDERBINEN, M., STEPHAN, R. & HÄCHLER, H. 2013. Higher-generation cephalosporin-resistant Escherichia coli in feral birds in Switzerland. *Int J Antimicrob Agents,* 41**,** 296-7.
